# Methods for Rapid Screening of Biologically Active Compounds Present in Plant-Based Extracts

**DOI:** 10.3390/molecules27207094

**Published:** 2022-10-20

**Authors:** Katarzyna Godlewska, Paweł Pacyga, Antoni Szumny, Anna Szymczycha-Madeja, Maja Wełna, Izabela Michalak

**Affiliations:** 1 Department of Pharmacology and Toxicology, The Faculty of Veterinary Medicine, Wrocław University of Environmental and Life Sciences, 50-375 Wrocław, Poland; 2 Department of Horticulture, The Faculty of Life Sciences and Technology, Wrocław University of Environmental and Life Sciences, 50-363 Wrocław, Poland; 3 Department of Thermodynamics and Renewable Energy Sources, Faculty of Mechanical and Power Engineering, Wrocław University of Science and Technology, 50-370 Wrocław, Poland; 4 Department of Chemistry, Faculty of Biotechnology and Food Science, Wrocław University of Environmental and Life Sciences, 50-375 Wrocław, Poland; 5 Department of Analytical Chemistry and Chemical Metallurgy, Faculty of Chemistry, Wrocław University of Science and Technology, 50-370 Wrocław, Poland; 6 Department of Advanced Material Technologies, Faculty of Chemistry, Wrocław University of Science and Technology, 50-372 Wrocław, Poland

**Keywords:** plant extracts, ultrasound-assisted extraction, bioactive compounds, screening tests, bioproducts

## Abstract

In recent years, there has been an increased interest in products of natural origin. The extraction procedure of bioactive compounds from plant matrices is a crucial step in the development of useful new bioproducts for everyday life. The utilisation of analyses enabling the rapid identification of the presence of a given group of compounds can be helpful in the early stages of the development of new products in order to save time and reduce costs. Within this article, we have presented a comparison of different, accessible methods for the identification of various bioactive compounds, e.g., saponins, carboxylic acids, oils and fats, proteins and amino acids, steroids, and alkaloids in plant-based extracts. Additionally, the multielemental composition of extracts was also examined. The applied methods allowed for confirmation of the presence of biologically active compounds in bio-products obtained by ultrasound-assisted extraction. At a later stage, these procedures should be supplemented by advanced analytical techniques in order to determine the plant chemicals’ content qualitatively and quantitatively.

## 1. Introduction

Plants are a rich source of phytochemicals that occur naturally in their leaves, stems, barks, flowers, seeds, fruits, and roots [1,2,3,4]. Nowadays, botanical products, containing a broad spectrum of compounds, are of great interest to scientists and consumers [2,5,6,7,8] due to their natural origin and availability [9]. The global market of plant extracts accounted for USD 30.8 billion in 2021 and is estimated to reach USD 55.3 billion by 2026 [10]. Medicinal and aromatic plants are widely used for aromatic, culinary, and therapeutic purposes as components of cosmetics, food, and medicinal and health products [5,6,8,11,12]. Moreover, plant-based extracts are incorporated into natural products, that are used in crop cultivation in order to increase their quality and tolerance to environmental stresses [13,14,15,16,17]. Primary plant constituents (e.g., sugars, proteins, amino acids, chlorophyll, and purines and pyrimidines of nucleic acids) are principally nutritional substances of plants and, in most cases, do not possess medicinal properties. Secondary plant constituents (secondary metabolites) such as alkaloids, carotenoids, flavonoids, glycosides, lignans, phenolic compounds, terpenoids, saponins, steroids, and tannins are accountable for multifarious biological, ecological, pharmacological, and toxicological activities [3,8,9,11,18,19]. Most of these secondary metabolites have a role in the defence against environmental factors (e.g., pollution, drought, UV light, pathogens) [3,19] and can be found in plants either permanently or only at certain stages of their growth [20]. Natural phytochemicals isolated from plants are considered to be attractive alternatives to synthetic chemicals [5,15,21,22,23]. From approximately 500,000 plant species, a mere 1–10% have been phytochemically examined [5,19,24]; therefore, there is a promising prospect to unveiling unique bioactive compounds [5]. These compounds are present in plants in trace quantities and are synthesised in specific cell types at various developmental stages; hence, they are hard to extract and purify [11]. The studies on bioactive plant compounds are dependent mainly on the selection of appropriate extraction methods, which are affected by different variables (e.g., matrix properties, pressure, solvents, temperature, time) [2]. Extraction is a crucial step in the analyses of plants and requires adequate preparation of raw materials (pre-washing, drying, grinding) to maintain the activity of the desired substances [8,9,25], as well as the selection of the appropriate solvent and standardised extraction procedure [9]. The choice of solvent type depends on the nature of the bioactive compound being targeted [25]. The extraction of hydrophilic compounds (e.g., flavonoids, organic acids, phenolic acids, sugars) requires the use of polar solvents (e.g., ethanol, methanol, or ethyl-acetate). In the extraction of lipophilic compounds (e.g., alkaloids, carotenoids, fatty acids, steroids, terpenoids, tocopherols), nonpolar solvents (e.g., dichloromethane, dichloromethane/methanol) should be utilised [2,8,25]. The selection of the solvent should consider the principles of Green Chemistry. For plant extraction, various methods are applied, for example: maceration, decoction, percolation, sonication, Soxhlet extraction, solid-phase micro-extraction, microwave-assisted extraction, ultrasound-assisted extraction, subcritical water extraction, supercritical fluid extraction, pressurised-liquid extraction, accelerated solvent extraction, and enzyme-assisted extraction [8,19,25,26]. For this study, we carried out experiments with the use of water as a solvent, because it dissolves a broad range of compounds and is nontoxic and non-flammable [9,26]. To disrupt plant cells, the ultrasound-assisted extraction was chosen, as it allows for a reduction in time and the amount of solvent [9]. Ultrasounds (above 20 kHz) enhance the solvent’s ability to penetrate the cells, increase the extraction yield [1], and lower solvent consumption [8].

The objective of this paper is to provide insight into the impact of ultrasound-assisted extraction on the phytochemical composition of plant-based extracts. Screening tests were conducted to detect the presence of metabolites (saponins, carboxylic acids, oil and fats, proteins and amino acids, steroids, terpenes, terpenoids, alkaloids) in 26 botanical extracts, as well as their multielemental composition, content of dry weight, and pH value.

## 2. Results

The applied methods were used to visually determine the presence of bioactive compounds in bio-extracts. They can be useful for quick screening; however, advanced analytical techniques are necessary to determine bio-extracts’ content qualitatively and quantitatively. To maintain clarity in the results, the following abbreviations were used: Alv L—aloe leaves; Am Fr—black chokeberry fruits; Arv H—common mugwort herb; Bv R—beetroot roots; Co F—common marigold flowers; Ea H—field horsetail herb; Ep F—purple coneflower flowers; Ep L—purple coneflower leaves; Hp H—St. John’s wort herb; Hr Fr—sea-buckthorn fruits; Lc S—red lentil seeds; Mc F—chamomile flowers; Ob H—basil herb; Pm H—broadleaf plantain herb; Poa H—common knotgrass herb; Ps S—pea seeds; Pta L—common bracken leaves; Sg L—giant goldenrod leaves; So R—comfrey roots; To F—common dandelion flowers; To L—common dandelion leaves; To R—common dandelion roots; Tp F—red clover flowers; Ur L—nettle leaves; Ur R—nettle roots; Vo R—valerian roots. The main factors in the selection of raw materials were their prevalence in Europe and the content of biologically active compounds. In all the tables in this article, the first tube from the left represents the control—aqueous extract without additives. Usually, in most cases, visual changes were observed immediately after mixing the extracts with appropriate reagents or after waiting a few minutes (up to 5 min).

### 2.1. Determination of Dry Weight

The highest dry weights of extracts were measured for extracts produced from black chokeberry fruits (Am Fr) and the roots of beetroot (Bv R), comfrey (So R,) and common dandelion (To R), whereas the lowest dry weights were found for the following biomasses: nettle roots (Ur R) and herbs such as common knotgrass (Poa H), St. John’s wort (Hp H) and common mugwort (Arv H). On average, the latter were three times lower than for those with the highest biomasses (Table 1).

### 2.2. Determination of pH

The pH of all examined extracts was acidic. Only for the extract from nettle leaves (Ur L) was the pH neutral. The highest acidic pH (about 6) was for extracts produced from purple coneflower leaves (Ep L), seeds of pea (Ps S), and red lentil (Lc S) and comfrey roots (So R). The lowest pH was measured for extracts obtained from fruits of sea-buckthorn (Hr Fr) and black chokeberry (Am Fr). In most cases, the pH was in the range of 4 to 5 (Table 1).

### 2.3. Determination of Saponins

The Froth test is used to detect saponins. A froth/foam formation indicates the presence of saponins (honeycomb froth) [25,27,28,29,30,31,32,33,34,35,36,37,38,39,40,41]. Saponins are amphiphilic glycosidic secondary metabolites with foaming properties, which are produced by plants [42]. The highest foam height was obtained for the extract from giant goldenrod leaves (Sg L) and seeds of pea (Ps S) and red lentil (Lc S). The results obtained for Sg L (*Solidago gigantea*) are in accordance with the results presented by Góral and Wojciechowski [42], in which the extract from the herb of *Solidago virgaurea* (woundwort) was indicated as saponin-rich. In the aforementioned work, soybean (*Glycine max* (L.) Merr.) was also proposed as one of the best sources of saponins; the soybean belongs to the same family (*Fabaceae*) as the pea (*Pisum sativum*) and red lentil (*Lens culinaris*) selected in the present study (Table 1).

### 2.4. Determination of Carboxylic Acids

NaHCO_3_ test–brisk effervescence indicates the presence of carboxylic acids [29]. In the present study, the NaHCO_3_ test did not indicate the presence of carboxylic acids in the examined extracts (Table 2).

### 2.5. Determination of Oils and Fats

(a) Saponification test–a soupy solution indicates the presence of oils and fats [27]. The application of this test to the examined extracts showed no presence of oils and fats (Table 2).
(b) Sudan test–a shining orange colour indicates the presence of fixed oil and fat [33]. The colour change in the extract was observed in many cases: Alv L (aloe leaves), Arv H (common mugwort herb), Co F (common marigold flowers), Ea H (field horsetail herb), Ep L (purple coneflower leaves), Hp H (St. John’s wort herb), Mc F (chamomile flowers), Ob H (basil herb), Pm H (broadleaf plantain herb), Poa H (common knotgrass herb), Pta L (common bracken leaves), Sg L (giant goldenrod leaves), So R (comfrey roots), To F (common dandelion flowers), To L (common dandelion leaves), To R (common dandelion roots), Tp F (red clover flowers), and Vo R (valerian roots). Generally, oils and fats were not present in extracts produced from fruits (e.g., chokeberry, sea-buckthorn), beetroot roots, representatives from the *Fabaceae* family such as as pea and red lentil, as well as nettle leaves and roots (Table 2).

The Sudan test was more effective at detecting oils and fats in aqueous extracts of higher plants than the saponification test.

### 2.6. Determination of Proteins and Amino Acids

There are many methods to visualise the presence of proteins and amino acids in plant extracts. These techniques include, for example:

(a) Biuret test (proteins)–a violet or pink colour precipitate indicates the presence of proteins [31,33,34,37,39]. This method showed the presence of proteins only in extracts produced from representatives of the *Fabaceae* family—pea and red lentil (Table 3).
(b) Ninhydrin test (amino acids)–a violet/purple/bluish colour indicates the presence of amino acids [27,28,31,37,38,40]. The change of the initial colour of extract into violet/purple/bluish was observed in almost all the examined extracts, aside from Alv L (aloe leaves), Am Fr (black chokeberry fruits), Ep F (purple coneflower flowers), Hp H (St. John’s wort herb), Hr Fr (sea-buckthorn fruits), Sg L (giant goldenrod leaves), and Ur L (nettle leaves) (Table 3).
(c) Millon’s test (proteins)–white precipitate, which turns red after heating, indicates the presence of proteins [28,37] and free amino acids [34,39]. This method turned out to be imprecise in determining the presence of proteins. White precipitation in the first stage was observed only for extracts from pea (Ps S) and red lentil (Lc S), but it did not turn into red, whereas red precipitate occurred after extract heating only in extracts produced from purple coneflower flowers (Ep F) and comfrey roots (So R) (Table 4).
(d) H_2_SO_4_ test (proteins)–white precipitate that turned to yellow after heating and orange after the addition of NH_4_OH revealed the presence of proteins [37]. The colour recognition of the precipitate was difficult due to the presence of diversified colours of extracts. Nonetheless, according to the expected observations, the following bioproducts that could contain some amounts of proteins can be mentioned: aloe leaves (Alv L), black chokeberry fruits (Am Fr), common marigold flowers (Co F), purple coneflower flowers (Ep F), purple coneflower leaves (Ep L), St. John’s wort herb (Hp H), red lentil seeds (Lc S), chamomile flowers (Mc F), common knotgrass herb (Poa H), pea seeds (Ps S), common bracken leaves (Pta L), giant goldenrod leaves (Sg L), comfrey roots (So R), common dandelion leaves (To L), common dandelion roots (To R), red clover flowers (Tp F).

### 2.7. Determination of Steroids, Terpenes, Terpenoids

The results of steroid, terpene, and terpenoid detection in plant extracts are presented in Table 5. The following methods were used to detect these secondary metabolites:

(a) Salkowski test–a red chloroform layer and greenish yellow fluorescence/green fluorescence of acid layer indicate the presence of steroids [34,36,39,40]. According to Shetty and Vijayalaxmi [27], a red precipitate can indicate the presence of steroids. A brown ring can also indicate the presence of steroids, and the appearance of bluish-brown ring can indicate the presence of phytosteroids [41]. Such observations were visible for the following extracts: beetroot roots (Bv R), common marigold flowers (Co F), chamomile flowers (Mc F), basil herb (Ob H), broadleaf plantain herb (Pm H), and pea seeds (Ps S).

A reddish-brown colouration of the interface indicated the presence of terpenoids [25,32,33,35,41]. The formation of two layers at the junction and a red colour in the lower layer indicates the presence of sterols, and a yellow colour in the upper layer can indicate the presence of triterpenoids [28]. No clear separation of the two phases was observed in tested plant extracts, but it can be assumed that triterpenoids were present in extracts produced from aloe leaves (Alv L), common marigold flowers (Co F), field horsetail herb (Ea H), sea-buckthorn fruits (Hr Fr), and common knotgrass herb (Poa H).

A golden yellow colour indicates the presence of triterpenes [38]. Based on the photos in the Table 5, it can be assumed that triterpenes were present in the following extracts: aloe leaves (Alv L), common marigold flowers (Co F), field horsetail herb (Ea H), sea-buckthorn fruits (Hr Fr), chamomile flowers (Mc F), common knotgrass herb (Poa H), red clover flowers (Tp F), nettle leaves (Ur L), and roots (Ur R). A solution colour change to cherry red indicates the presence of phytosterols [29]. On the basis of the colours of plant extracts presented in Table 5, most of them contained phytosterols.

(b) Acetic acid and H_2_SO_4_ test–a blue-green ring indicates the presence of terpenoids [31]. In the examined extracts, there was no blue-green ring. This method did not allow for the detection of terpenoids in plant extracts.
(c) Liebermann–Burchard test–a pink or red colour indicates the presence of steroids [25]. This was only observed for the extracts produced from black chokeberry fruits (Am Fr) and purple coneflower flowers (Ep F). According to Jayapriya et al. [41], a green colour indicates the presence of phytosterols. In the present study, these compounds can be detected in extracts from chamomile flowers (Mc F) and from nettle roots (Ur R). In the examined extracts, there was no green precipitate, which can indicate the presence of steroids [27]. In tested extracts we did not observe (1) the change of red colour through blue to green can indicate the presence of steroids [33]; (2) the formation of a reddish-violet colour at the junction can indicate the presence of steroids, triterpenoids, and cardiac glycosides [36]; and (3) a brown ring appears at the junction of two layers—a green colour in the upper layer can indicate the presence of sterols, and the formation of deep-red colour in the lower layer indicates the presence of triterpenoids [28].

### 2.8. Determination of Alkaloids

The observations for the alkaloids’ detection in plant extracts are summarised in Table 6 and Table 7.

(a) Hager’s test–a yellow precipitate indicates the presence of alkaloids [28,33,34,37]. Such a precipitate was observed only in extracts from pea seeds (Ps S) and red lentil seeds (Lc S)–Table 6.
(b) Tannic acid test–a buff colour indicates the presence of alkaloids [28]. A more intensive colour of extract was observed only for black chokeberry fruits (Am Fr), beetroot roots (Bv R), giant goldenrod leaves (Sg L), comfrey roots (So R), and valerian roots (Vo R)–Table 6.
(c) Mayer’s test (1)–a pale, white, creamy precipitate indicates the presence of alkaloids [27,28,29,35,37,38]. The application of this test didn’t indicate the presence of alkaloids in examined plant extracts–Table 6.
(d) Mayer’s test (2)–a green colour or white precipitate indicates the presence of alkaloids [41]. An opalescence or yellowish precipitate indicates the presence of alkaloids [30,36,39]. This technique was more sensitive for the detection of alkaloids in the extracts than the previous one. A white precipitate was observed in extract from pea seeds (Ps S). Yellow-orange fine precipitate was detected in more extracts, for example, from common mugwort herb (Arv H), common marigold flowers (Co F), St. John’s wort herb (Hp H), basil herb (Ob H), pea seeds (Ps S), common bracken leaves (Pta L), comfrey roots (So R), and common dandelion leaves (To L)–Table 6.
(e) Dragendroff’s test (1)–the presence of alkaloids can be indicated by a reddish-brown precipitate [33,43], an orange-brown precipitate [29,30], and an orange precipitate [27]. In the present study, the precipitate described above was observed in extracts produced from common marigold flowers (Co F), red lentil seeds (Lc S), and pea seeds (Ps S)–Table 7.
(f) Dragendroff’s test (2) –indicates the presence of alkaloids when an orange or red precipitate [35,40] or an orange-brown precipitate [34,36,39] occurs. The plant extracts that indicated the presence of alkaloids were common mugwort herb (Arv H), common marigold flowers (Co F), purple coneflower flowers (Ep F) and leaves (Ep L), basil herb (Ob H), pea seeds (Ps S), comfrey roots (So R), and common dandelion leaves (To L)–Table 7.
(g) Wagner’s test–a reddish-brown precipitate indicates the presence of alkaloids [27,28,29,33,35,37]. In the examined extracts, their colour was generally changed into reddish brown, but no evident precipitate was present–Table 7.

### 2.9. Determination of Multielemental Composition

The selected raw materials (RMs) and their extracts (Es) differed significantly in terms of elemental composition (Table 8, Table 9 and Table 10).

For instance, amidst microelements, the highest content of Al in RMs was found in nettle roots (Ur R), common marigold flowers (Co F), and So R (comfrey roots), and the content ranged from 831 to 587 mg·kg^−1^. In the case of extracts, the greatest level of Al was present in the product based on So R (6.08 mg·L^−1^), followed by Vo R (2.92 mg·L^−1^) and Alv L (2.20 mg·L^−1^). The lowest amounts occurred in pea seeds (Ps S) (0.943 mg·kg^−1^) and their extract (0.023 mg·L^−1^). The purple coneflower leaves (Ep L) and their extract contained the highest amount of B (222 mg·kg^−1^ and 7.86 mg·L^−1^, respectively). The lowest amount of this element was observed in red lentil seeds (Lc S) (6.56 mg·kg^−1^), Ps S (7.08 mg·kg^−1^), and So R (9.25 mg·kg^−1^). The vast majority of extracts contained less than 1 mg·L^−1^ of boron. The amount of Fe ranged from 19.4 mg·kg^−1^ (for black chokeberry fruits, Am Fr) to 720 mg·kg^−1^ (for common marigold flowers, Co F). Most of the extracts contained less than 1 mg·L^−1^ of iron, with the exception of aloe leaves (Alv L, 5.21 mg·L^−1^) and So R (comfrey roots, 4.85 mg·L^−1^) along with nettle roots (Ur R, 1.11 mg·L^−1^), (Co F, 1.27 mg·L^−1^), basil herb (Ob H, 1.34 mg·L^−1^), pea seeds (Ps S, 1.44 mg·L^−1^), red lentil seeds (Lc S, 1.58 mg·L^−1^), and valerian roots (Vo R, 1.58 mg·L^−1^). The amount of Zn among the raw materials was diverse and varied from 5.41 mg·kg^−1^ (for black chokeberry fruits, Am Fr) to 50.1 mg·kg^−1^ (common knotgrass herb, Poa H). The generality of extracts contained less than 1 mg·L^−1^ of zinc, and the lowest value was noted for black chokeberry fruits (Am Fr, 0.093 mg·L^−1^). The exceptions were purple coneflower flowers (Ep F, 3.37 mg·L^−1^), common bracken leaves (Pta L, 1.71 mg·L^−1^), St. John’s wort herb (Hp H, 1.12 mg·L^−1^), and pea seeds (Ps S, 1.07 mg·L^−1^). It can be seen that the highest content of a given microelement in the biomass did not always indicate its highest content in the extract.

In the case of macroelements, the content of Ca was the highest in nettle leaves (Ur L, 45,964 mg·kg^−1^) and purple coneflower leaves (Ep L, 40,254 mg·kg^−1^), whereas it was the lowest in red lentil seeds (Lc S, 300 mg·kg^−1^) and pea seeds (Ps S, 642 mg·kg^−1^). The extract based on purple coneflower leaves (Ep L, 1234 mg·L^−1^) was also characterised by a high content of calcium, as was the one obtained from aloe leaves (Alv L, 1193 mg·L^−1^). Lentil seed extract (Lc S, 7.43 mg·L^−1^) and sea-buckthorn fruit extract (Hr Fr, 10.5 mg·L^−1^) contained low levels of calcium. The content of K in the extracts mostly depended on its amount in raw materials: more potassium was transferred to the extract while more of it was in the biomass. For example, raw materials, such as purple coneflower flowers (Ep F, 39,187 mg·kg^−1^), common dandelion leaves (To L, 39,140 mg·kg^−1^), and basil herb (Ob H, 33,427 mg·kg^−1^) and their extracts (Ep F, 1414 mg·L^−1^; To L, 1319 mg·L^−1^; Ob H, 1218 mg·L^−1^) contained the highest content of potassium. In turn, red lentil seeds (Lc S, 8702 mg·kg^−1^), sea-buckthorn fruits (Hr Fr, 8467 mg·kg^−1^), black chokeberry fruits (Am Fr, 8118 mg·kg^−1^), St. John’s wort herb (Hp H, 7608 mg·kg^−1^) and their extracts (Lc S, 171 mg·L^−1^; Hr Fr, 234 mg·L^−1^; Am Fr, 206 mg·L^−1^; Hp H, 283 mg·L^−1^) were characterised by one of the lowest amounts of potassium. A similar trend can be observed in the magnesium content. The biomasses that contained the highest amount of Mg were: aloe leaves, basil herb, and nettle leaves (Alv L, 7472 mg·kg^−1^; Ob H, 6978 mg·kg^−1^; Ur L, 8611 mg·kg^−1^), and their extracts contained 319 mg·L^−1^, 217 mg·L^−1^, and 206 mg·L^−1^, respectively. Among the sources (biomasses and extracts), those with the lowest magnesium content can be mentioned: black chokeberry fruits (Am Fr, 795 mg·kg^−1^; 13.8 mg·L^−1^), red lentil seeds (Lc S, 781 mg·kg^−1^; 17.2 mg·L^−1^), and sea-buckthorn fruits (Hr Fr, 684 mg·kg^−1^; 16.7 mg·L^−1^). The sodium content ranged from 20 mg·kg^−1^ in black chokeberry fruits (Am Fr) and 0.760 mg·L^−1^ in purple coneflower leaves’ extract (Ep L) to 8327 mg·kg^−1^ and 442 mg·L^−1^ in broadleaf plantain herb (Pm H). The amount of P was the highest in chamomile flowers (Mc F, 4960 mg·kg^−1^) and common dandelion flowers (To F, 4853 mg·kg^−1^), and the lowest was in black chokeberry fruits (Am Fr, 1331 mg·kg^−1^). The extracts of common dandelion flowers (To F, 182 mg·L^−1^) contained the highest level of potassium, whereas sea-buckthorn fruits (Hr Fr, 18.0 mg·L^−1^) and nettle leaves (Ur L, 12.6 mg·L^−1^) contained the lowest. The amount of S ranged from 455 mg·kg^−1^ and 4.53 mg·L^−1^ in black chokeberry fruits (Am Fr) and their extract to 9683 mg·kg^−1^ and 453 mg·L^−1^ in broadleaf plantain herb (Pm H) and its extract.

Most of the raw materials contained less than 0.7 mg·kg^−1^ of Cd, with the exception of the common mugwort herb (Arv H), which included 1.84 mg·kg^−1^ of this element. The cadmium content was below the limit of detection (LOD) in the vast majority of extracts; the highest amount was in common mugwort herb extract (Arv H) (0.011 mg·L^−1^). The content of Cr ranged from 0.269 mg·kg^−1^ in common dandelion leaves (To L) to 6.08 mg·kg^−1^ in common knotgrass herb (Poa H). In the extracts, the concentration was from 0.0015 mg·L^−1^ in common mugwort herb (Arv H) to 0.248 mg·L^−1^ in common dandelion flowers (To F) (except in purple coneflower flowers (Ep F), which amounted 1.84 mg·L^−1^). The nickel quantity in raw materials was diversified: some contained a lower amount of Ni (such as beetroot roots (Bv R)–0.702 mg·kg^−1^), whereas others had higher amounts (e.g., red lentil seeds (Lc S)–10.8 mg·kg^−1^, pea seeds (Ps S)–11.8 mg·kg^−1^, nettle roots (Ur R)–10.7 mg·kg^−1^). Extracts based on purple coneflower flowers (Ep F), purple coneflower leaves (Ep L), and common dandelion flowers (To F) contained the highest levels of nickel (4.92 mg·L^−1^, 1.27 mg·L^−1^, 1.08 mg·L^−1^, respectively), whereas others contained less than 0.362 mg·L^−1^ (present in common marigold flowers, Co F). In the majority of cases, the lead quantity was below the limit of detection. The conducted analysis revealed that only three raw materials (common marigold flowers (Co F), common bracken leaves (Pta L), and purple coneflower leaves (Ep L)) contained above 2.00 mg·kg^−1^ of this heavy metal (2.83 mg·kg^−1^, 2.74 mg·kg^−1^, and 2.60 mg·kg^−1^, respectively), and five of them contained more than 1.00 mg·kg^−1^ (broadleaf plantain herb (Pm H)–1.82 mg·kg^−1^, nettle leaves (Ur R)–1.49 mg·kg^−1^, valerian roots (Vo R)–1.41 mg·kg^−1^, comfrey roots (So R)–1.34 mg·kg^−1^, red clover flowers (Tp F)–1.05 mg·kg^−1^).

## 3. Discussion

Plants, due to the content of bioactive compounds and secondary metabolites (e.g., alkaloids, flavonoids, phenolics, steroids, tannins, and terpenoids) provide significant ecological, pharmacological, and economical benefits [2]. At present, approximately 80% of the global population employs plant extracts for basic healthcare needs [25,44]. Natural products (NPs), being a source of complex mixtures of compounds, exhibit miscellaneous biological activities, including antibiotic, anticancer, antidiarrheal, antidiabetic, antihypertensive, anti-inflammatory, antimicrobial, antioxidant, antiparasitic, and hypoglycemic [25,44,45], as well as analgesic and wound-healing properties [25]. NPs (as pure compounds or as standardised extracts) offer endless possibilities for novel drug inventions by virtue of their peerless availability of chemical variety [25]. Natural products are successfully used in cosmetic, phytotherapy, and food-additive applications [44]. Plant secondary metabolites (e.g., terpenes, flavonoids, phenolic and polyphenolic compounds, nitrogen-containing and sulphur-containing compounds) also play a significant ecological role in crop defence (against, for example, bacteria, fungi, herbivores, plants, viruses), attraction and stimulation (e.g., nutrient sequestration, pollination), and counter abiotic stresses [18].

Saponins, a group of structurally varied molecules, are widely distributed surface-active glycosides with specific foaming properties [46,47,48,49]. Triterpenoid saponins are common in cultivated plants (soybeans, peas, beans, lucerne, tea, spinach, sunflower), whereas steroidal saponins mostly occur in herbs (soapbark, soapberry, soap root, soapwort) [47,48]. These compounds exhibit numerous properties that encompass both favourable and adverse impacts on human health, pesticidal, insecticidal, and molluscicidal activity, allelopathic action, antinutritional effects, sweetness and bitterness, and as phyto-protectants (against microbes and herbivores) [47,48,49]. Saponins are used in numerous applications, for example, medicine (due to their antimicrobial, anticancer, anti-inflammatory activity and beneficial role in cardiovascular disease), and in the pharmaceutical industry, they are used in the semi-synthesis of steroidal drugs. They are also utilised in the production of cosmetics (as stabilisers of emulsions, foam intensifications), food (as foaming agents in beverages and confectionery), (3) detergents, and (4) fire extinguishers [46,49,50]. Based on our primary research on the determination of saponins using the Froth test, the best source of these compounds was the extract obtained from giant goldenrod leaves (Sg L), whereas they were virtually not present in black chokeberry fruit (Am Fr) and sea-buckthorn fruit (Hr Fr) extracts.

Carboxylic acids or organic acids, possessing the carboxyl functional group [51,52,53], are grouped as aromatic, saturated, or unsaturated acids. They are intermediates in the degradation pathways of amino acids, carbohydrates, and fats [52]. These acids are widely spread in nature [52,53] or can be obtained in laboratory [52]. Due to their multiple functions, they are used for various applications in agriculture, the food industry, pharmacy, or medicine [51,52,54]. Carboxylic acids are used in the production of a broad range of products, e.g., adhesives, biopolymers, coatings, drugs, polymers, foods and beverages (as acidulants, antioxidants, emulsifiers, flavours, or preservatives) [52,54]. The quantitative assessment of organic acids levels in human body fluids can supply an initial identification of diverse ailments. They also find an application in pharmaceuticals (as solubilisers, prodrugs, bioprecursors, and pharmacophores) [52,55], cosmetics (to clean pores, improve the skin texture, or in acne treatment) [52,56], as well in the production of air fresheners, deodorants, and perfumes [52,57]. As presented in the Results section, the method of carboxylic acids’ extraction with the use of the NaHCO_3_ test did not allow for an indication of the presence of these chemicals in the examined plant extracts.

Fats and oils are triglycerides (solid, semi-solid, or liquid) that are suppliers of energy and nutrients [58,59,60,61]. Plants constitute an essential resource of these compounds from nature [62]. Their roles in the human body include, e.g., hormonal effects, protection of delicate organs, carrying soluble vitamins, or sensory palatability [61], and in food systems, they provide flavours and textures and improve aeration and moisture retention, therefore being effective cooking in frying [59,61]. In the pharmaceutical and cosmetic industry, oils are used as excipients, coadjuvants, transdermal carriers, and skin emolliency agents [63]. Plant oils can be used as well as a liquid fuel, which could be beneficial in view of rising petroleum prices and environmental concerns [62,64]. They can be applied in the production of lubricants and inks [62], soaps, and corrosion inhibitors [65]. The Sudan test allows for the observation of the presence of fixed oil and fat in numerous botanical extracts, for instance, in products based on aloe leaves (Alv L), common mugwort herb (Arv H), common marigold flowers (Co F), field horsetail herb (Ea H), purple coneflower leaves (Ep L), St. John’s wort herb (Hp H), chamomile flowers (Mc F), basil herb (Ob H), broadleaf plantain herb (Pm H), common knotgrass herb (Poa H), common bracken leaves (Pta L), giant goldenrod leaves (Sg L), comfrey roots (So R), common dandelion flowers, leaves, and roots (To F, To L, To R), red clover flowers (Tp F), as well as valerian roots (Vo R). Fixed oils and fats were not noted in the extracts obtained, for example, from black chokeberry fruits (Am Fr), sea-buckthorn fruits (Hr Fr), beetroot roots (Bv R), pea seeds (Ps S), red lentil seeds (Lc S), and nettle leaves and roots (Ur L, Ur R).

Plants, due to the large quantities of carbohydrates that make up their structure, contain lower amounts of protein compared to animal cells [66]. However, the vegetal food production has a lower environmental footprint than animal husbandry. Beyond 60% of the proteins necessary for human growth and development originate from plant resources [67]. Amino acids (AA) are components of proteins [66,67] and affect plethora of plant physiological processes (e.g., growth and development, resistance to environmental stresses, control of intracellular pH) [66,68]. On the basis of their synthesis in humans, they are classified as essential (synthesised only by plants) and non-essential (synthesised by plants and human) amino acids [67,69,70]. These compounds affect human physiological function and are used in medicine and nutrition [65]. Amino acids play a crucial role in metabolic processes and in the transport and storage of nutrients. The proper composition of AA is a requisite to correcting metabolic dysfunction and protecting from multiple diseases (e.g., arthritis, diabetes, insomnia, obesity) [67,70]. The application of the biuret test to detect proteins allowed for the confirmation of their presence in the extracts of pea seeds (Ps S) and red lentil seeds (Lc S), and the use of Millon’s test confirmed their presence in extracts based on purple coneflower flowers (Ep F) and comfrey roots (So R). On the other hand, the use of the sulphuric acid test enabled the observation of proteins in extracts produced from aloe leaves (Alv L), black chokeberry fruits (Am Fr), common marigold flowers (Co F), purple coneflower flowers (Ep F), purple coneflower leaves (Ep L), St. John’s wort herb (Hp H), red lentil seeds (Lc S), chamomile flowers (Mc F), common knotgrass herb (Poa H), pea seeds (Ps S), common bracken leaves (Pta L), giant goldenrod leaves (SgL), comfrey roots (So R), common dandelion leaves (To L), common dandelion roots (To R), and red clover flowers (Tp F). In most cases, the amino acids (marked by the use of the ninhydrin test) were present in plant extracts, with the exception of products based on aloe leaves (Alv L), black chokeberry fruits (Am Fr), purple coneflower flowers (Ep F), St. John’s wort herb (Hp H), sea-buckthorn fruits (Hr Fr), giant goldenrod leaves (Sg L), and nettle leaves (Ur L).

Steroids are secondary metabolites produced by animals, plants, and microorganisms [71,72], and those found in plants can be grouped into (1) compounds with physiological function in the plant itself (e.g., hormones, pheromones), (2) allelochemical compounds related to animal hormones, and (3) allelochemical compounds with protective functions [73,74,75,76]. They are used in agriculture [77], cosmetics, herbal medicine, and nutrition [78]. They play a key role in the pharmacological activities of medicines [72]. Phytosterols are considered to be crucial dietary agents for lowering blood cholesterol, as well as improving immune function [74], protection against cancer [74,76], and cardiotonic [78] and antioxidant enzymes activity [74], as well as containing antibacterial properties [79]. They are used in food production (margarine and milk derivatives). Through the manipulation of phytosterol profiles, the level of other metabolites (improper for consumption or industrial processing) can be influenced [74]. Terpenoids are a large and structurally varied class of plant natural products that occur in many plants [80,81,82] and are essential to their survival [80]. More than 50 000 different structures of these compounds have been reported to date [83]. Terpenoids are active compounds that possess miscellaneous properties beneficial to humans [80]. They are widely applied in the industrial sector as flavours, fragrances, and spices, as well as in cosmetics, medicine, nutraceuticals, and perfumery [80,81]. They exhibit a broad spectrum of activities, for example: antimicrobial, anticancer, antifoaming, carminative, hepatoprotective, neuroprotective, anti-inflammatory, and antinociceptive actions [81,84]. The implementation of the Salkowski test confirmed that the following extracts—beetroot roots (Bv R), common marigold flowers (Co F), chamomile flowers (Mc F), basil herb (Ob H), broadleaf plantain herb (Pm H), and pea seeds (Ps S)—contained steroids, whereas triterpenoids were present in the extracts produced from aloe leaves (Alv L), common marigold flowers (Co F), field horsetail herb (Ea H), sea-buckthorn fruits (Hr Fr), and common knotgrass herb (Poa H). Triterpenes were present in the extracts obtained from aloe leaves (Alv L), common marigold flowers (Co F), field horsetail herb (Ea H), sea-buckthorn fruits (Hr Fr), chamomile flowers (Mc F), common knotgrass herb (Poa H), red clover flowers (Tp F), nettle leaves (Ur L), and roots (Ur R). Most of the plant extracts contained phytosterols. The acetic acid and H_2_SO_4_ test did not allow for the detection of terpenoids in plant extracts. The steroids, verified by the Liebermann–Burchard test, were only observed in extracts produced from black chokeberry fruits (Am Fr) and purple coneflower flowers (Ep F), whereas extracts from chamomile flowers (Mc F) and nettle roots (Ur R) contained phytosterols.

Alkaloids are a large group of approximately 27,000 organic nitrogen-containing compounds that constitute one of the most abundant classes of metabolites [85,86,87]. Most alkaloids are biosynthetically derived from amino acids (e.g., lysine, ornithine, phenylalanine, tryptophan, and tyrosine [87,88]) and are often classified, based on their molecular skeletons, into: pyridine, tropane, quinoline, isoquinoline, phenanthrene, phenylethylamine, indole, purine, imidazole, and terpenoid groups of alkaloids [85,89]. These compounds are normally produced by plants (especially flowering) as toxic substances [86]. They take part in the improvement of reproductive rates, among other methods, by the incrementation of defences against plant growth stressors (biotic and abiotic) [85]. They are also of great importance in human life [88]. It is estimated that over than 17,000 of them exhibit pharmacological activities [86], in particular: anticancer, antibacterial, antiviral, anti-inflammatory, analgesic, antiasthmatic, antiarrhythmic, antipyretics, antihypertensive, antihyperglycemic, and antihypertensive effects [87,90]. They are frequently used in clinical practice, for example: morphine as a narcotic analgesic, codeine to reduce coughing, vinblastine to treat cancer, and berberine hydrochloride as an antibacterial agent [87]. The major attention of alkaloid research is focused in pharmacology/pharmacy (17.53%), plant sciences (13.24%), and medicinal chemistry (9.96%) [88]. Due to diversified activities, their production, extraction, and processing are one of the key research directions and developments [88,91]. The applied methods showed different sensitivities of the alkaloid detection in plant extracts. The preparations that could be considered a potential source of these compounds include, among others, common mugwort herb (Arv H), common marigold flowers (Co F), red lentil seeds (Lc S), basil herb (Ob H), pea seeds (Ps S), common bracken leaves (Pta L), comfrey roots (So R), and common dandelion leaves (To L).

For the proper growth of plants, seventeen elements are required (i.e., C, H, O, N, P, K, Ca, Mg, S, Fe, Zn, Mn, B, Cu, Mo, Cl, Ni) [92,93]. The carbon, hydrogen, and oxygen are uptaken from carbon dioxide and water, while the remaining elements, termed mineral nutrients, are from the soil [92,94]. The mineral nutrients are divided into two groups: macronutrients (consisting of elements in large supply, such as Ca, K, Mg, N, P, S) and micronutrients (usually needed in small amounts, such as B, Cl, Cu, Fe, Mn, Mo, Ni, Zn) [92]. The demand of living organisms for micronutrients is low, yet despite this, micronutrients are crucial for the essential cell functions and can significantly affect the growth and quality of plants, as well as the health of animals and humans [94,95]. Sufficient intake of these elements may help in the prevention or treatment of varied ailments (e.g., arterial hypertension and bone demineralization) and hidden hunger. Plants are a good source of these minerals and should be an integral part of the human diet [94]. However, the mineral content of plants depends on their species and parts as well as growth conditions, in particular, their geographic location, soil constitution, water source, irrigation, and type of fertilisers [96]. For this reason, proper attempts are necessary to overcome the shortages of micronutrients. The results of this study revealed that the botanical extracts could be considered a source of micro- and macroelements. On average, the macronutrients were present in the greatest amounts in the extracts based on aloe leaves (Alv L), purple coneflower leaves (Ep L), broadleaf plantain herb (Pm H), nettle leaves (Ur L), basil herb (Ob H), and common dandelion leaves (To L), whereas micronutrients were present in products obtained from common marigold flowers (Co F), purple coneflower flowers (Ep F), basil herb (Ob H), common dandelion flowers (To F), aloe leaves (Alv L), valerian roots (Vo R), common knotgrass herb (Poa H), giant goldenrod leaves (Sg L), and common dandelion leaves (To L).

In the present research, the selected methods allowed for the detection of the biologically active compounds in plant materials. Ultrasound-assisted extraction can be used to easily extract these natural chemicals, which are generally recognised as safe. Phytochemicals elicit many beneficial health effects in man and animals and will continue to play an important role in numerous therapies and as preventive agents against diseases, as well as in other industries.

## 4. Materials and Methods

### 4.1. Chemicals and Reagents

The following chemicals were used for analysis of the content of bioactive compounds: sodium bicarbonate (Honeywell), potassium sodium tartrate tetrahydrate (Merck), potassium hydroxide (Merck), phenolphthalein (Avantor), Sudan III (Waldeck), sodium hydroxide (Avantor), copper(II) sulphate (Avantor), mercuric chloride (Avantor), mercuric nitrate (Sigma Aldrich), mercurous nitrate (Alfa Aesar), nitric acid (Merck), sulphuric acid (Avantor), ammonium hydroxide (Supelco), ninhydrin (Sigma Aldrich), acetone (Stanlab), chloroform (Avantor), acetic acid (Supelco), acetic anhydride (Avantor), picric acid (Sigma Aldrich), tannic acid (Alfa Aesar), mercury(II) chloride (Avantor), bismuth subnitrate (Honeywell), glacial acetic acid (Supelco), potassium iodide (Sigma Aldrich), hydrochloric acid (Avantor), iodine (Sulpeco), multi-elemental stock ICP standard solution (no. XVI, Merck), single ICP stocks of As, Hg, P, S, and Se (Merck).

### 4.2. Plant Materials Used for Extraction of Compounds

Raw materials (with abbreviations) selected for the preparation of extracts used in this study included:

Alv L–aloe leaves, *Aloe vera* (L.) Burm. f.;
Am Fr–black chokeberry fruits, *Aronia melanocarpa* (Michx.) Elliott;
Arv H–common mugwort herb, *Artemisia vulgaris* L.;
Bv R–beetroot roots, *Beta vulgaris* L.;
Co F–common marigold flowers, *Calendula officinalis* L.;
Ea H–field horsetail herb, *Equisetum arvense* L.;
Ep F–purple coneflower flowers, *Echinacea purpurea* (L.) Moench;
Ep L–purple coneflower leaves, *Echinacea purpurea* (L.) Moench;
Hp H–St. John’s wort herb, *Hypericum perforatum* L.;
Hr Fr–sea-buckthorn fruits, *Hippophae rhamnoides* L.;
Lc S–red lentil seeds, *Lens culinaris* Medik.;
Mc F–chamomile flowers, *Matricaria chamomilla* L.;
Ob H–basil herb, *Ocimum basilicum* L.;
Pm H–broadleaf plantain herb, *Plantago major* L.;
Poa H–common knotgrass herb, *Polygonum aviculare* L.;
Ps S–pea seeds, *Pisum sativum* L.;
Pta L–common bracken leaves, *Pteridium aquilinum* (L.) Kuhn;
Sg L–giant goldenrod leaves, *Solidago gigantea* Ait.;
So R–comfrey roots, *Symphytum officinale* L.;
To F–common dandelion flowers, *Taraxacum officinale* (L.) Weber ex F.H. Wigg.;
To L–common dandelion leaves, *Taraxacum officinale* (L.) Weber ex F.H. Wigg.;
To R–common dandelion roots, *Taraxacum officinale* (L.) Weber ex F.H. Wigg.;
Tp F–red clover flowers, *Trifolium pratense* L.;
Ur L–nettle leaves, *Urtica dioica* L.;
Ur R–nettle roots, *Urtica dioica* L.;
Vo R–valerian roots, *Valeriana officinalis* L.

### 4.3. Extraction

Bioproducts were obtained by means of ultrasound-assisted extraction (UAE) using the homogeniser UP 50 H (Hielscher Ultrasonics GmbH, Teltow, Germany). Dried and ground (500 μm mesh size) biomass was well-mixed in a glass beaker with deionised water (ratio 1:20 *w*/*v*), left for maceration (30 min, room temperature), sonicated (30 min), and centrifuged (4500 rpm, 10 min, Heraeus Megafuge 40, rotor TX-750, Thermo Scientific, Waltham, MA, USA). The obtained supernatants were used for analyses.

### 4.4. Analyses of Extracts

#### 4.4.1. Determination of Dry Weight

Extracts were dried at 50 °C to the constant weight.

#### 4.4.2. Determination of pH

The pH of extracts was measured using a pH meter (Mettler Toledo, Seven Multi).

#### 4.4.3. Determination of Saponins

Aqueous test–distilled water (5 mL) was added to the extract (1 mL), and the resulting mixture was shaken vigorously for 15 min (Shatty and V 2012). The height of the foam (which lasted for 5 min) was measured.

#### 4.4.4. Determination of Carboxylic Acid

NaHCO_3_ test–the extract (2 mL) was treated with NaHCO_3_ (5%, 2 mL) [29].

#### 4.4.5. Determination of Oils and Fats

(a) Saponification test–KOH (0.1 N, 10 drops) and phenophthalein idndicator (5 drops) were added to the extract (2 mL), and then the mixture was heated in a water bath (70 °C, 1.5 h) [27].
(b) Sudan test–Sudan III solution (few drops) (0.5 g of dye was diluted in 100 mL of 99% isopropanol) was added to the extract (2 mL) [33].

#### 4.4.6. Determination of Proteins and Amino Acids

(a) Biuret test (for proteins)–biuret reagent (a few drops) was added to the extract (2 mL) [34]. The reagent was prepared by dissolving pentavalent copper sulphate (1.5 g) and potassium sodium tartrate (6.0 g) in distilled water (500 mL). Sodium hydroxide (2 M, 375 mL) was added, and the volumetric flask was filled up with distilled water (up to 1000 mL).
(b) Millon’s test (for proteins)–Millon’s reagent (2 mL) was added to the extract (2 mL) and heated in a water bath (5 min) [37]. The Millon’s reagent was made by dissolving mercuric nitrate (160 g) and mercurous nitrate (160 g) in concentrated nitric acid (400 mL) and made up to 1000 mL with distilled water.
(c) H_2_SO_4_ test (for proteins)–concentrated H_2_SO_4_ (1 mL) was added to the extract (3 mL). Subsequently, NH_4_OH (1 mL) was appended [37].
(d) Ninhydrin test (for amino acids)–a ninhydrin solution (2 mL, 0.2% in acetone) was added to the extract (2 mL), and the solution was boiled in a water bath (10 min) [31].

#### 4.4.7. Determination of Steroids

(a) Salkowski test–chloroform (2 mL) and concentrated H_2_SO_4_ (2 mL) were added to the extract (2 mL) [34].
(b) Acetic acid and H_2_SO_4_ test (terpenoids)–the extract (2 mL) was treated with acetic acid (2 mL) and sulphuric acid (1 mL) [31].
(c) Liebermann–Burchard test–chloroform (1 mL), acetic anhydride (2 mL), and concentrated sulphuric acid (2 drops) were added to the extract (1 mL) [25].

#### 4.4.8. Determination of Alkaloids

(a) Hager’s test–Hager’s reagent (5 drops) (1% solution of picric acid in water) was added to the extract (2 mL) [37].
(b) Tannic acid test–tannic acid solution (1 mL, 10% in water) was added to the extract (2 mL) [28].
(c) Mayer’s test (1)–Mayer’s reagent (10 drops) was added on the sides of the test tube containing the extract (3 mL) [35]. The Mayer’s reagent was prepared by mixing two separately prepared aqueous solutions: mercuric chloride (1.36 g in 60 mL of distilled water) and potassium iodide (5 g in 10 mL of distilled water). The solutions were combined in a 100 mL volumetric flask and filled up to the mark with distilled water.
(d) Mayer’s test (2)–concentrated hydrochloric acid (2 mL) and Mayer’s reagent (8 drops) were added to the extract (2 mL) [41].
(e) Dragendroff’s test (1)–Dragendroff’s reagent (1 mL) was added to the extract (2 mL) [43]. The Dragendroff’s reagent was prepared by mixing (in ratio 1:1) solution A, prepared by dissolving bismuth nitrate (0.85 g) in water (40 mL) and acetic acid (10 mL), and solution B was prepared by dissolving potassium iodine (8 g) in water (20 mL). Then, 5 mL of each solution was transferred to a 100 mL volumetric flask, mixed with glacial acetic acid (20 mL), diluted with water to volume, and mixed [97,98,99].
(f) Dragendroff’s test (2)–HCl (1 mL) and Dragendroff’s reagent (0.5 mL) were added to the extract (2.5 mL) [40].
(g) Wagner’s test–Wagner’s reagent (5 drops) was added to the extract (2 mL) [29].

#### 4.4.9. Determination of Multielemental Composition

Sample preparation: the total concentrations of elements in the raw materials and extracts were determined by ICP OES following the microwave-assisted closed-vessel digestion. Plant biomasses (0.250 g) and extracts (5.0 mL) were placed into PTFE vessels and poured over with 5.0 mL of concentrated HNO_3_. The digestion in the microwave reaction system employed a six-step microwave-assisted heating program (190 °C, 60 min). The obtained remnants were cooled down to room temperature and quantitatively transferred into 30-mL polypropylene containers (Equimed, Wrocław, Poland), diluted with deionised water to 25.0 g, and kept at 4 °C until measurement. Analyses were made in triplicate (*n* = 3). The digestion of samples was carried out using a Multiwave PRO microwave reaction system (Anton Paar GmbH, Austria; 24HVT50 rotor; 50 mL PTFE-TFM pressure-activated-venting vessels).

Instrumentation: the spectrometric analyses were conducted with the use of bench-top simultaneous optical emission Ar-ICP spectrometer (Agilent, model 720) with the torch in the axial alignment. The apparatus consists of a four-channel peristaltic pump, a high-resolution Echelle-type polychromator with temperature-controlled optics, and a VistaChip II CCD detector. To sustain and operate the plasma, a standard, one-piece, quartz torch with an injector tube (2.4 mm ID) was used. For the pneumatic nebulisation of the sample solution, a glass single-pass cyclonic spray chamber (Agilent) combined with a concentric OneNeb nebuliser (Agilent) was applied. The operating settings were as follows: the RF power was 1.2 kW; gas flow rates (in L·min^−1^) were 15.0 (plasma), 1.5 (auxiliary), and 0.75 (nebuliser); the sample flow rate was 0.75 mL·min^−1^, and the instrument stabilisation and sample uptake delays were 15 s, rinsed and replicated times 10 and 1 s (three replicates). The analytical lines selected for measurements were as follows: 396.1 nm (Al), 188.9 nm for (As), 249.7 (B), 455.5 nm (Ba), 317.9 nm (Ca), 214.4 nm (Cd), 267.7 nm (Cr), 327.3 nm (Cu), 238.2 nm (Fe), 253.6 nm (Hg), 766.4 nm (K), 285.2 nm (Mg), 257.6 nm (Mn), 589.5 nm (Na), 231.6 (Ni), 213.6 nm (P), 220.6 nm (Pb), 181.9 nm (S), 196.0 nm (Se), 407.7 nm (Sr), and 213.8 (Zn). To quantify the content of the elements, external calibration curves (six points, concentration range from 0 to 5 mg·L^−1^) were applied.

## 5. Conclusions

Plant-based extracts will continue to be essential and renewable sources of biologically active compounds in most sectors of industry, starting from foods, through cosmetics and agriculture agents, to medicine products and drugs. The presented techniques for the detection of valuable compounds are accessible and inexpensive and provide a quick answer as to the composition of the obtained bio-products. However, continuous progress in analytical techniques, especially chromatographics, should allow for the identification of as-yet unidentified plant chemicals and open up new perspectives for their utilisation in the near future.

## Figures and Tables

**Table 1:** The pH value (*n* = 3), dry weight (*n* = 3), and the presence of saponins in botanical extracts.

Extract	Dry Weight, %	pH	Saponins-Froth Test (Foam Height)
**Alv L**	0.42 ± 0.02	4.26 ± 0.06	1.4 cm
**Am Fr**	0.62 ± 0.04	3.92 ± 0.11	0.1 cm
**Arv H**	0.20 ± 0.01	4.97 ± 0.04	0.6 cm
**Bv R**	0.61 ± 0.03	5.00 ± 0.04	0.6 cm
**Co F**	0.38 ± 0.03	4.18 ± 0.10	0.4 cm
**Ea H**	0.25 ± 0.03	5.27 ± 0.04	0.2 cm
**Ep F**	0.43 ± 0.03	4.77 ± 0.09	1.0 cm
**Ep L**	0.38 ± 0.05	6.35 ± 0.12	0.2 cm
**Hp H**	0.20 ± 0.02	4.06 ± 0.05	1.4 cm
**Hr Fr**	0.39 ± 0.04	2.32 ± 0.13	not present
**Lc S**	0.32 ± 0.04	5.96 ± 0.12	2.0 cm
**Mc F**	0.23 ± 0.03	4.41 ± 0.05	0.2 cm
**Ob H**	0.27 ± 0.02	5.30 ± 0.11	0.4 cm
**Pm H**	0.36 ± 0.02	4.70 ± 0.03	0.4 cm
**Poa H**	0.16 ± 0.03	4.68 ± 0.14	0.4 cm
**Ps S**	0.27 ± 0.03	6.11 ± 0.11	2.2 cm
**Pta L**	0.24 ± 0.02	5.09 ± 0.10	1.0 cm
**Sg L**	0.31 ± 0.02	4.78 ± 0.03	4.2 cm
**So R**	0.61 ± 0.04	5.63 ± 0.14	0.4 cm
**To F**	0.36 ± 0.03	4.06 ± 0.08	0.1 cm
**To L**	0.36 ± 0.03	5.04 ± 0.12	0.2 cm
**To R**	0.63 ± 0.06	5.14 ± 0.12	0.2 cm
**Tp F**	0.22 ± 0.02	4.90 ± 0.05	1.0 cm
**Ur L**	0.23 ± 0.05	7.14 ± 0.65	0.2 cm
**Ur R**	0.17 ± 0.02	5.43 ± 0.12	0.4 cm
**Vo R**	0.41 ± 0.06	4.41 ± 0.07	0.4 cm

**Table 2:** The presence of carboxylic acid, oils, and fats in botanical extracts.

Method	Carboxylic Acids–NaHCO_3_ Test			Oils and Fats–Saponification Test			Oils and Fats–Sudan Test		
Extract	Observations	Result	Photograph	Observations	Result	Photograph	Observations	Result	Photograph
**Alv L**	Colour change of the solution to orange-brick-red	–	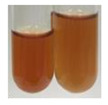	Colour change of the solution to brown	–	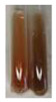	Colour change of the solution to orange	+	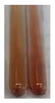
**Am Fr**	Colour change of the solution brownish grey	–	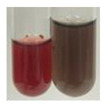	Colour change of the solution to claret	–	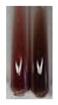	No changes were observed	–	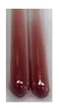
**Arv H**	Colour change of the solution to brownish yellow	–	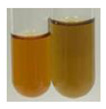	Colour change of the solution to brick red	–	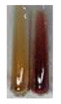	Colour change of the solution to orange	+	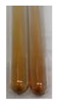
**Bv R**	No changes were observed	–	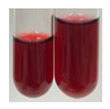	Colour change of the solution to brownish yellow	–	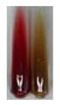	No changeswere observed	–	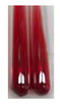
**Co F**	Colour change of the solution to bright orange	–	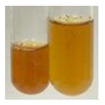	Colour change of the solution to orange, a fine precipitation formed	–	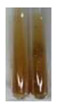	Colour change of the solution to orange	+	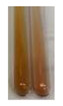
**Ea H**	Colour change of the solution to bright orange	–	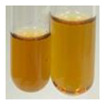	Colour change of the solution to brownish yellow; a fine precipitation formed	–	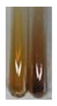	Colour change of the solution to orange	+	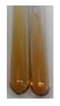
**Ep F**	Colour change of the solution to brown	–	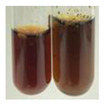	Colour change of the solution to brown	–	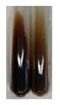	No changes were observed	–	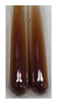
**Ep L**	Colour change of the solution to olive green	–	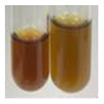	Colour change of the solution to brown; a fine precipitation formed	–	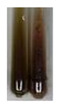	Tonal colour change of the solution	+	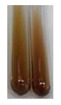
**Hp H**	Colour change of the solution to orange yellow	–	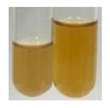	Colour change of the solution to brick-red	–	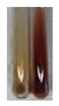	Colour change of the solution to red- orange	+	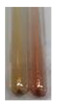
**Hr Fr**	Colour change of the solution to lemon yellow	–	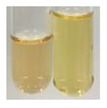	Colour change of the solution to cloudy yellow	–	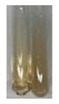	Colour change of the solution to bright orange	–	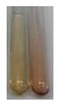
**Lc S**	Colour change of the solution to flesh-coloured/colourless	–	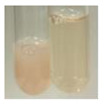	Colour change of the solution to pink	–	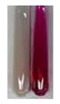	Colour change of the solution to bright pink	–	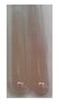
**Mc F**	No changes were observed	–	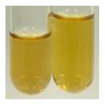	Colour change of the solution to orange	–	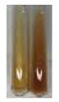	Colour change of the solution to orange	+	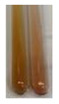
**Ob H**	Colour change of the solution to brown-orange	–	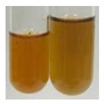	Colour change of the solution to brown; a fine precipitation formed	–	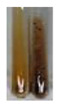	Colour change of the solution to orange	+	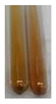
**Pm H**	Colour change of the solution to yellow-orange-brown	–	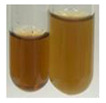	Colour change of the solution to brown	–	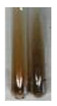	Colour change of the solution to amber- orange	+	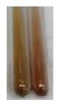
**Poa H**	Colour change of the solution to bright yellow	–	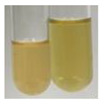	Colour change of the solution to raspberry	–	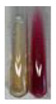	Colour change of the solution to orange	+	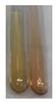
**Ps S**	Colour change of the solution to light straw	–	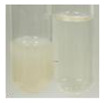	Colour change of the solution to pink	–	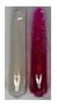	Colour change of the solution to light pink	–	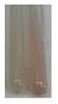
**Pta L**	Colour change of the solution to orange	–	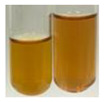	Colour change of the solution to intense pink	–	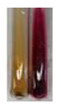	Colour change of the solution to orange	+	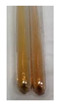
**Sg L**	Colour change of the solution to brown-orange	–	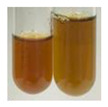	Colour change of the solution to brown	–	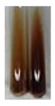	Colour change of the solution to orange	+	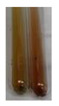
**So R**	Colour change of the solution to brown-orange	–	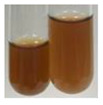	Colour change of the solution to brick-red	–	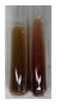	Colour change of the solution to amber- orange	+	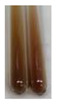
**To F**	Colour change of the solution to bright orange	–	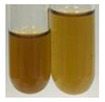	No changeswere observed	–	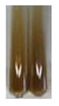	Colour change of the solution to amber with orange glow	+	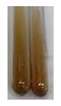
**To L**	Colour change of the solution to light brown	–	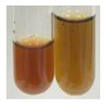	Colour change of the solution to brown	–	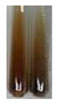	Colour change of the solution to orange	+	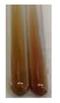
**To R**	Colour change of the solution to light straw	–	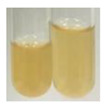	Colour change of the solution orange-red	–	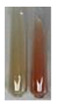	Colour change of the solution to pinkish-orange	+	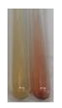
**Tp F**	Colour change of the solution to light yellow	–	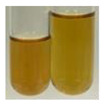	Colour change of the solution to orange	–	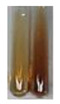	Colour change of the solution to orange	+	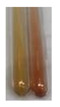
**Ur L**	Colour change of the solution to yellow-olive	–	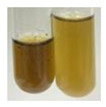	Colour change of the solution to pink	–	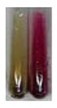	Colour change of the solution to amber	–	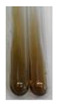
**Ur R**	Colour change of the solution to light yellow	–	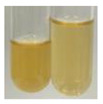	Colour change of the solution to red-pink	–	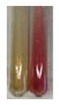	Colour change of the solution to orange	+	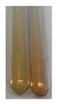
**Vo R**	Colour change of the solution to brown-orange	–	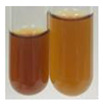	Colour change of the solution to brown	–	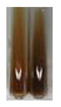	Colour change of the solution to amber- orange	+	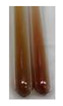

**Table 3:** The presence of proteins and amino acids in botanical extracts—biuret and ninhydrin tests.

Method	Biuret Test			Ninhydrin Test		
Extract	Observations	Result	Photograph	Observations	Result	Photograph
**Alv L**	Colour change of the solution to yellow	–	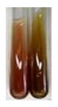	Colour change of the solution to light orange	–	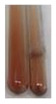
**Am Fr**	Colour change of the solution on the sides of the test tube to brown	–	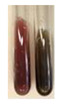	Tonal colour change of the solution to light red	-	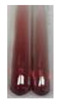
**Arv H**	Colour change of the solution to yellow-green	–	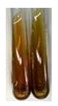	Colour change of the solution to purple	+	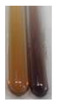
**Bv R**	Colour change of the solution to orange	–	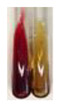	Colour change of the solution to purple	+	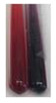
**Co F**	Colour change of the solution to dirty-yellow	–	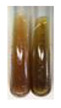	Colour change of the solution to purple	+	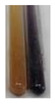
**Ea H**	Colour change of the solution on the sides of the test tube to green-yellow	–	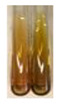	Colour change of the solution to purple	+	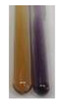
**Ep F**	Colour change of the solution on the sides of the test tube to yellow-green	–	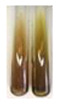	Colour change of the solution to amber-yellow	–	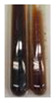
**Ep L**	Colour change of the solution on the sides of the test tube to yellow-green	–	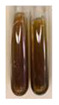	Colour change of the solution to purple-brown	+	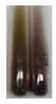
**Hp H**	Colour change of the solution to yellow	–	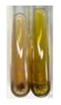	No changes were observed	–	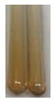
**Hr Fr**	Colour change of the solution on the sides of the test tube to yellow	–	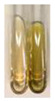	Colour change of the solution to light brown	–	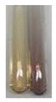
**Lc S**	Colour change of the solution to violet	+	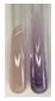	Colour change of the solution to purple; a white precipitation formed	+	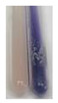
**Mc F**	Colour change of the solution to yellow	–	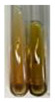	Colour change of the solution to purple	+	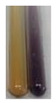
**Ob H**	Colour change of the solution to yellow	–	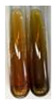	Colour change of the solution to purple	+	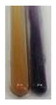
**Pm H**	Colour change of the solution to yellow-green	–	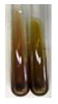	Colour change of the solution to purple	+	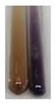
**Poa H**	Colour change of the solution to green-yellow	–	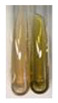	Colour change of the solution to purple	+	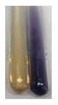
**Ps S**	Colour change of the solution to violet	+	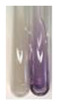	Colour change of the solution to purple; a white precipitation formed	+	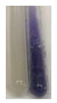
**Pta L**	Colour change of the solution to copper	–	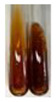	Colour change of the solution to purple	+	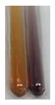
**Sg L**	Colour change of the solution to brown- yellow	–	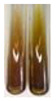	Colour change of the solution to orange-yellow	–	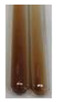
**So R**	Colour change of the solution to dirty yellow	–	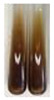	Colour change of the solution to purple-brown	+	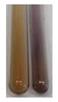
**To F**	Colour change of the solution to yellow	–	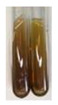	Colour change of the solution to purple	+	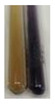
**To L**	Colour change of the solution to yellow	–	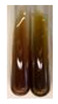	Colour change of the solution to purple	+	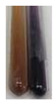
**To R**	Colour change of the solution to green	–	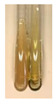	Colour change of the solution to purple	+	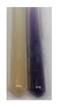
**Tp F**	Colour change of the solution to yellow	–	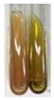	Colour change of the solution to purple	+	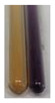
**Ur L**	Colour change of the solution on the sides of the test tube to green	–	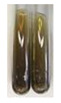	Colour change of the solution to orange-red	–	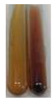
**Ur R**	Colour change of the solution on the sides of the test tube to green	–	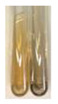	Colour change of the solution to purple	+	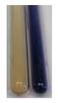
**Vo R**	Colour change of the solution to dirty yellow	–	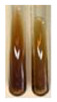	Colour change of the solution to purple	+	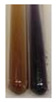

**Table 4:** The presence of proteins and amino acids in botanical extracts–Millon’s and H_2_SO_4_ tests.

Method	Millon’s Test			H_2_SO_4_ Test		
Extract	Observations	Result	Photograph	Observations	Result	Photograph
**Alv L**	Before boiling: red solution After boiling: orange- yellow solution	Before: – After: –	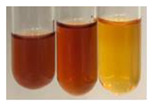	After addition of H_2_SO_4_: the colour changed to orange and brown. After boiling with H_2_SO_4_: the colour changed to orange, red-black, and precipitation formed. After addition of H_2_SO_4_ and NH_4_OH: the colour changed to brown-red, orange, brown.	1: – 2: +/– 3: –	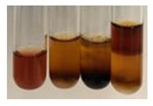
**Am Fr**	Before boiling: orange-yellow solution After boiling: lemon solution	Before: – After: –	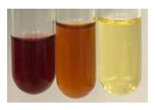	After addition of H_2_SO_4_: the colour changed to red and black. After boiling with H_2_SO_4_: the colour changed to red and black, and a fine precipitation formed. After addition of H_2_SO_4_ and NH_4_OH: the colour changed to orange, brown, and red, plus fine precipitation	1: – 2: +/– 3: +/–	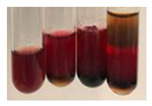
**Arv H**	Before boiling: orange solution After boiling: lemon solution	Before: – After: –	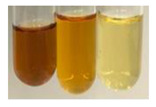	After addition of H_2_SO_4_: the colour changed to orange-red, brown, and black. After boiling with H_2_SO_4_: the colour changed to brown-red and black. After addition of H_2_SO_4_ and NH_4_OH: the colour changed to olive-green, red, brown, and black.	1: – 2: – 3: –	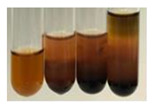
**Bv R**	Before boiling: orange solution After boiling: lemon solution	Before: – After: –	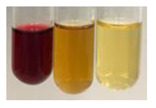	After addition of H_2_SO_4_: the colour changed to burgundy, orange, and black. After boiling with H_2_SO_4_: the colour changed to brown and black. After addition of H_2_SO_4_ and NH_4_OH: the colour changed to red and black.	1: – 2: – 3: –	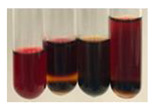
**Co F**	Before boiling: orange solution + orange-brown precipitation formed After boiling: yellow-lemon solution + a white precipitation formed	Before: – After: –	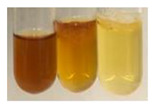	After addition of H_2_SO_4_: the colour changed to amber-orange and red. After boiling with H_2_SO_4_: the colour changed to red-brown and black, and a brown precipitation formed. After addition of H_2_SO_4_ and NH_4_OH: the colour changed to yellow, orange, red, and black, plus a colourless glow	1: – 2: +/– 3: –	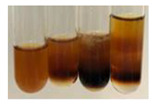
**Ea H**	Before boiling: orange solution After boiling: lemon solution	Before: – After: –	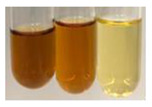	After addition of H_2_SO_4_: the colour changed to orange-brown. After boiling with H_2_SO_4_: the colour changed to brown and black. After addition of H_2_SO_4_ and NH_4_OH: the colour changed to brown, orange, and yellow.	1: – 2: – 3: –	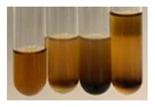
**Ep F**	Before boiling: red colour and brown precipitation After boiling: yellow solution and red precipitation	Before: – After: +	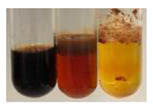	After addition of H_2_SO_4_: the colour changed to red- orange, formation of a yellow-black ring. After boiling with H_2_SO_4_: the colour changed to black and red. After addition of H_2_SO_4_ and NH_4_OH: the colour changed to brown, dirty pink-red, and black.	1: + 2: +/– 3: +/–	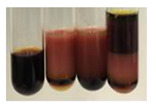
**Ep L**	Before boiling: orange-red solution After boiling: yellow solution	Before: – After: –	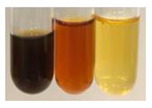	After addition of H_2_SO_4_: the colour changed to “cappuccino” colour, black. After boiling with H_2_SO_4_: the colour changed to brown and black. After addition of H_2_SO_4_ and NH_4_OH: the colour changed to green, brown, and black.	1: + 2: +/– 3: +/–	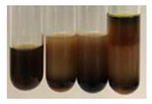
**Hp H**	Before boiling: orange solution and orange precipitation After boiling: lemon solution	Before: – After: –	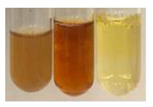	After addition of H_2_SO_4_: the colour changed to orange-red along with a fine precipitation; a black colour developed. After boiling with H_2_SO_4_: the colour changed to red, with red-brown precipitation. After addition of H_2_SO_4_ and NH_4_OH: the colour changed to orange, brown-red, and dark maroon.	1: + 2: +/– 3: +/–	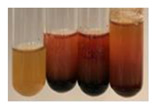
**Hr Fr**	Before boiling: orange solution After boiling: yellow solution	Before: – After: –	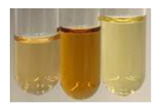	After addition of H_2_SO_4_: the colour changed to yellow-orange and brown. After boiling with H_2_SO_4_: the colour changed to orange, brown, and black. After addition of H_2_SO_4_ and NH_4_OH: the colour changed to yellow, light lemon, and brown.	1: – 2: – 3: –	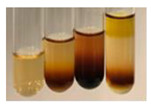
**Lc S**	Before boiling: yellow-orange solution and precipitation of a white colour After boiling: light-lemon solution + formation of a white precipitation	Before: + After: –	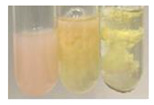	After addition of H_2_SO_4_: the colour changed to orange-brown, pink precipitation formation. After boiling with H_2_SO_4_: the colour changed to brown-orange, pink precipitation formation. After addition of H_2_SO_4_ and NH_4_OH: the colour changed to light lemon, plus a fine precipitation and red-orange colour.	1: + 2: +/– 3: +/–	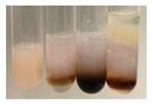
**Mc F**	Before boiling: orange solution After boiling: lemon solution	Before: – After: –	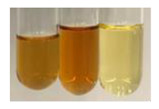	After addition of H_2_SO_4_: the colour change to light orange and brown. After boiling with H_2_SO_4_: the colour changed to brown, dark brown. After addition of H_2_SO_4_ and NH_4_OH: the colour changed to amber, orange, red-brown, plus a colourless glow.	1: + 2: – 3: –	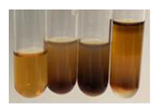
**Ob H**	Before boiling: orange solution After boiling: lemon solution	Before: – After: –	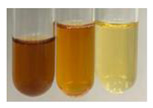	After addition of H_2_SO_4_: the colour changed to cloudy orange and brown. After boiling with H_2_SO_4_: the colour changed to brown and black. After addition of H_2_SO_4_ and NH_4_OH: the colour changed to amber, orange.	1: + 2: – 3: –	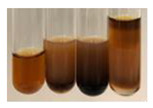
**Pm H**	Before boiling: orange solution After boiling: lemon solution	Before: – After: –	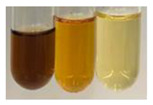	After addition of H_2_SO_4_: the colour changed to brown-red and dark maroon. After boiling with H_2_SO_4_: the colour changed to brown, blood-brown, and black. After addition of H_2_SO_4_ and NH_4_OH: the colour changed to brown, orange, and black.	1: – 2: – 3: –	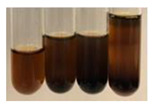
**Poa H**	Before boiling: yellow- orange solution After boiling: lemon solution	Before: – After: –	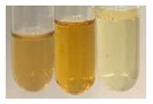	After addition of H_2_SO_4_: the colour changed to yellow and orange. After boiling with H_2_SO_4_: the colour changed to brown, orange, and red. After addition of H_2_SO_4_ and NH_4_OH: the colour changed to red, orange, yellow, and dark yellow.	1: +/– 2: +/– 3: +/–	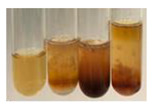
**Ps S**	Before boiling: yellow- colourless solution + precipitation formed After boiling: light-lemon solution + precipitation of a white precipitation	Before: + After: –	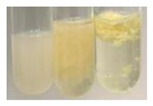	After addition of H_2_SO_4_: the colour changed to light pink and red-brown. After boiling with H_2_SO_4_: the colour changed to light pink and brown. After addition of H_2_SO_4_ and NH_4_OH: the colour changed to lemon, white precipitation, red-brown.	1: + 2: +/– 3: +/–	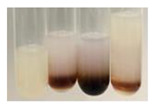
**Pta L**	Before boiling: orange solution After boiling: lemon solution	Before: – After: –	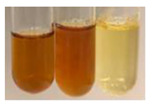	After addition of H_2_SO_4_: the colour changed to yellow-orange and black. After boiling with H_2_SO_4_: the colour changed to orange; a fine precipitation formed, black colour. After addition of H_2_SO_4_ and NH_4_OH: the colour changed to orange, yellow, and black, plus orange Precipitation.	1: + 2: +/– 3: +/–	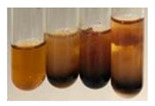
**Sg L**	Before boiling: orange-red solution After boiling: yellow solution	Before: – After: –	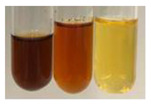	After addition of H_2_SO_4_: the colour changed to amber, yellow/green, and black. After boiling with H_2_SO_4_: formation of olive/brown Precipitation; the colour change to black and yellow-brown. After addition of H_2_SO_4_ and NH_4_OH: the colour changed to brown, yellow-orange, plus a colourless glow.	1: + 2: +/– 3: +/–	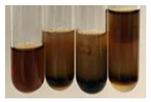
**So R**	Before boiling: yellow-lemon solution + brown precipitation After boiling: yellow-lemon solution + precipitation of a brick-red colour	Before: – After: +	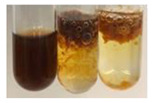	After addition of H_2_SO_4_: formation of a jelly-like consistency; the colour changed to orange/brown/black After boiling with H_2_SO_4_: formation of a jelly-like consistency; the colour changed to brown/black. After addition of H_2_SO_4_ and NH_4_OH: the colour changed to brown, and black, plus a colourless glow.	1: + 2: +/– 3: +/–	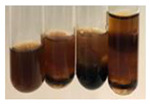
**To F**	Before boiling: orange solution + formation of an amber-coloured precipitation After boiling: yellow-lemon solution	Before: – After: –	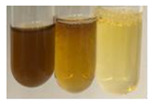	After addition of H_2_SO_4_: the colour changed to brown and black. After boiling with H_2_SO_4_: the colour change to dark brown plus a black glow After addition of H_2_SO_4_ and NH_4_OH: the colour changed to orange, amber, and blood-brown.	1: – 2: – 3: –	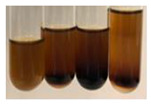
**To L**	Before boiling: orange solution + formation of a fine precipitation After boiling: yellow solution	Before: – After: –	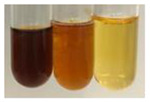	After addition of H_2_SO_4_: formation of brown-orange and fine precipitation. After boiling with H_2_SO_4_: the colour changed to brown plus a black glow. After addition of H_2_SO_4_ and NH_4_OH: the colour changed to black and orange, plus a colourless glow.	1: + 2: +/– 3: +/–	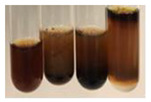
**To R**	Before boiling: cloudy orange solution After boiling: light lemon solution	Before: – After: –	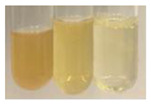	After addition of H_2_SO_4_: the colour changed to light yellow and black. After boiling with H_2_SO_4_: the colour changed to orange plus fine precipitation, black-brown colour. After addition of H_2_SO_4_ and NH_4_OH: the colour changed to yellow, lemon, black, and yellow/colourless.	1: – 2: +/– 3: –	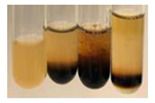
**Tp F**	Before boiling: orange solution After boiling: lemon solution	Before: – After: –	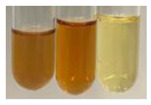	After addition of H_2_SO_4_: the colour changed to red- orange and brown. After boiling with H_2_SO_4_: the colour changed to brown-red, black colour. After addition of H_2_SO_4_ and NH_4_OH: the colour changed to yellow-orange, pale orange, and brown.	1: +/– 2: +/– 3: –	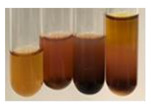
**Ur L**	Before boiling: light-orange solution After boiling: lemon solution	Before: – After: –	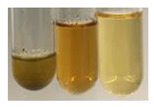	After addition of H_2_SO_4_: the colour changed to amber, orange, and colourless. After boiling with H_2_SO_4_: the colour changed to brown- yellow with an amber glow. After addition of H_2_SO_4_ and NH_4_OH: the colour changed to yellow, orange, and amber.	1: – 2: – 3: –	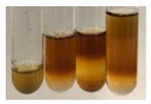
**Ur R**	Before boiling: light-yellow solution After boiling: light lemon solution	Before: – After: –	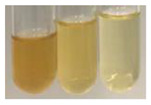	After addition of H_2_SO_4_: the colour changed to yellow-orange. After boiling with H_2_SO_4_: the colour changed to orange, yellow, amber colour. After addition of H_2_SO_4_ and NH_4_OH: the colour changed to yellow, pale yellow, and colourless solution.	1: – 2: – 3: –	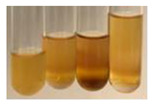
**Vo R**	Before boiling: orange solution After boiling: yellow solution	Before: – After: –	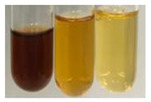	After addition of H_2_SO_4_: the colour changed to brown-red and black. After boiling with H_2_SO_4_: the colour changed to brown and black. After addition of H_2_SO_4_ and NH_4_OH: the colour changed to orange-brown.	1: – 2: – 3: –	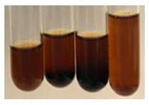

**Table 5:** The presence of steroids, terpenes, and terpenoids in botanical extracts.

Method	Salkowski Test			Acetic acid and H_2_SO_4_ Test			Liebermann-Burchard Test		
Extract	Observations	Result	Photograph	Observations	Result	Photograph	Observations	Result	Photograph
**Alv L**	The separation of 4 phases was observed: orange, colourless, amber and colourless.	+ triterpenoids, triterpenes	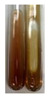	The formation of 3 phases: orange, white precipitate, orange-brown.	–	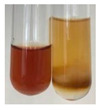	A yellow colour change of the solution was observed with precipitation	–	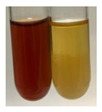
**Am Fr**	The separation of 3 phases was observed: dirty blood, translucent, and dark maroon.	+ phytosterols	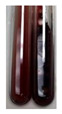	The formation of 2 phases: red, black.	–	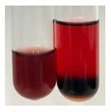	The separation of 2 red and colourless phases was observed	+ steroids	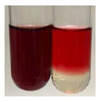
**Arv H**	The separation of 3 phases was observed: brick, colourless, and black.	+ phytosterols	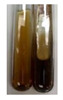	The formation of 2 phases: orange, brown.	–	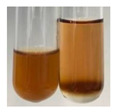	The separation of 2 phases: light orange and colourless.	–	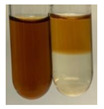
**Bv R**	The separation of 4 phases was observed: blood, colourless, black, and slightly yellow.	+ steroids, phytosterols	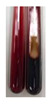	The formation of 3 phases: dark raspberry, orange, and black and red.	–	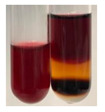	The separation of 3 phases was observed: orange, yellow, and colourless.	–	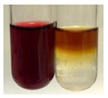
**Co F**	The separation of 4 phases was observed: amber, slightly yellow, brown, and yellow.	+ steroids, triterpenoids, triterpenes	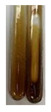	The formation of 2 phases: orange and red-black.	–	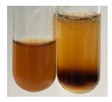	The separation of 3 phases was observed: orange, lemon, and colourless. A slight precipitation was noticed in the highest phase.	–	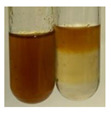
**Ea H**	Two phases were distinguished: yellow and dark amber.	+ triterpenoids, triterpenes	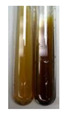	The formation of 2 phases: yellow and red-brown.	–	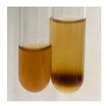	The separation of 2 phases with a light precipitate was observed: yellow and colourless.	–	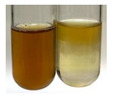
**Ep F**	The separation of 2 phases was observed: dirty brown and colourless.	+ phytosterols	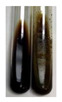	The appearance of a black ring was observed; the solution turned red.	–	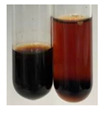	The separation of 2 phases was observed: colourless and brick-red.	+ steroids	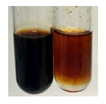
**Ep L**	The separation of 2 phases was observed: black and colourless.	–	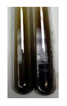	The formation of 3 phases: orange, cloudy light brown, black.	–	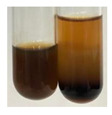	Precipitation of a yellow-orange solid was observed.	–	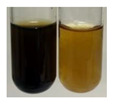
**Hp H**	The separation of 3 phases was observed: brick, colourless, and black.	+ phytosterols	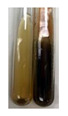	The formation of 3 phases: red-salmon, orange, black, and red.	–	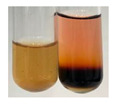	The separation of 2 phases was observed: salmon and colourless.	–	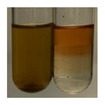
**Hr Fr**	The separation of 4 phases was observed: colourless twice, yellow, and brick.	+ triterpenoids, triterpenes	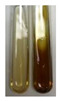	The formation of 2 phases: a light lemon and an orange-red ring.	–	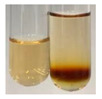	The separation of 2 phases was observed: slightly yellow and colourless.	–	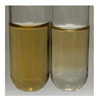
**Lc S**	Separation of 2 phases was observed: colourless and red- orange.	–	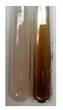	The formation of 2 phases: light powder pink and a red-brown ring.	–	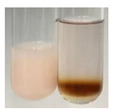	Discolouration of the solution was observed, with a slight salmon glow.	–	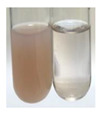
**Mc F**	The separation of 3 phases was observed: maroon, yellow, and orange-yellow.	+ steroids, triterpenes	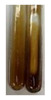	The formation of 2 phases: yellow and orange.	–	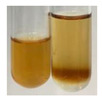	Colour change of the solution to light green was observed.	+ phytosterols	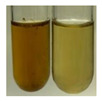
**Ob H**	The separation of 2 phases was observed: dark brown and slightly yellow.	+ steroids, phytosterols	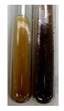	The formation of 2 phases: orange and red-brown.	–	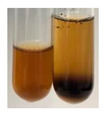	A colour change to orange-yellow in the solution and precipitation were observed.	–	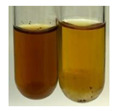
**Pm H**	The separation of 2 phases was observed: dark brown and slightly lemon.	+ steroids, phytosterols	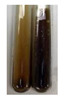	The formation of 2 phases: brown solution and brown ring.	–	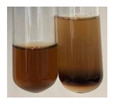	The separation of 3 phases was observed: orange, salmon with a delicate precipitation, and colourless.	–	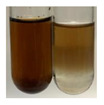
**Poa H**	The separation of 4 phases was observed: orange, colourless, brick-red, and light red.	+ triterpenoids, triterpenes	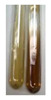	The formation of 3 phases: bright orange, orange, and dark orange.	–	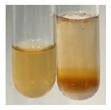	The separation of 2 phases was observed: orange and colourless.	–	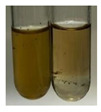
**Ps S**	The separation of 4 phases was observed: dirty beige, colourless, brick-red, and slightly yellow.	+ steroids	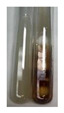	The formation of 2 phases: light powder pink and red-brown ring.	–	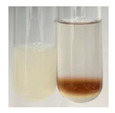	The colour of the solution changed to a delicate powder pink, and the formation of a weak precipitate was observed.	–	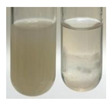
**Pta L**	The separation of 3 phases was observed: black, colourless, and brick-orange	+ phytosterols	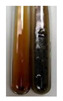	The formation of 2 phases: yellow, black and red.	–	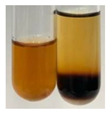	The separation of 3 phases was observed: orange, yellow and colourless.	–	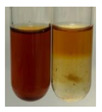
**Sg L**	Development of a yellow ring was observed.	+ phytosterols	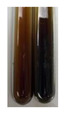	The formation of 2 phases: orange and red-black.	–	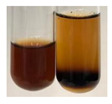	The separation of 3 phases was observed: orange, lemon, and colourless.	–	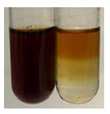
**So R**	The separation of 3 phases was observed: brick-red with a gelatinous consistency, colourless, and black.	+ phytosterols	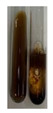	The formation of 2 phases: orange with dispersed precipitation and a black ring.	–	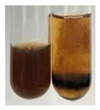	The separation of 2 phases was observed: amber and colourless.	–	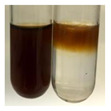
**To F**	The separation of 3 phases was observed: amber, colourless, and black.	–	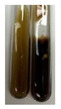	The formation of 2 phases: orange and reddish brown ring.	–	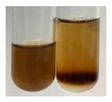	The separation of 2 phases was observed: orange and colourless.	–	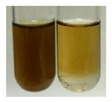
**To L**	The separation of 2 phases was observed: dirty brown and colourless.	+ phytosterols	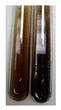	The formation of 2 phases: orange and red ring.	–	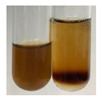	A slight precipitate was observed at the bottom, and the solution turned orange.	–	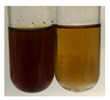
**To R**	The separation of 3 phases was observed: light brown, colourless, and black.	–	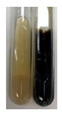	The formation of 2 phases: light yellow and black and red.	–	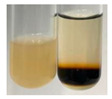	The colour change of the solution to lemon was observed.	–	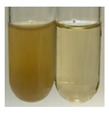
**Tp F**	The separation of 2 phases was observed: amber and yellow.	+ steroids, triterpenes	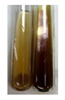	The formation of 2 phases: orange and red-brown.	–	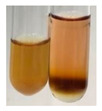	The colour of the solution changed to a delicate salmon orange colour with precipitation.	–	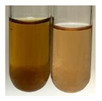
**Ur L**	The separation of 2 phases was observed: dark amber and colourless.	+ triterpenes	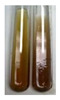	The formation of 2 phases: dirty orange and bright orange.	–	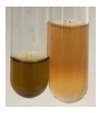	The formation of a white precipitate and a change of colour of the solution to yellow-straw were observed.	–	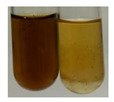
**Ur R**	The separation of 2 phases was observed: colourless and light amber.	+ triterpenes	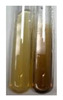	The formation of 3 phases: light lemon, yellow and orange.	–	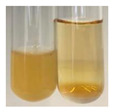	The separation of 2 phases was observed: light green and colourless.	+ phytosterols	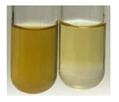
**Vo R**	The separation of 3 phases was observed: brown, colourless, and dark brown.	+ terpenoids	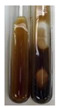	The formation of 2 phases: orange and red-black.	–	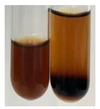	The separation of 2 phases was observed: orange and colourless.	–	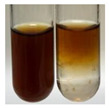

**Table 6:** The presence of alkaloids in botanical extracts–Hager’s, Tannic acid, Mayer’s tests.

Method	Hager’s Test			Tannic Acid Test			Mayer’s Test (1)			Mayer’s Test (2)		
Extract	Observations	Result	Photograph	Observations	Result	Photograph	Observations	Result	Photograph	Observations	Result	Photograph
**Alv L**	Colour change of the solution to orange-yellow	–	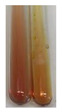	Cappuccino- coloured precipitation	–	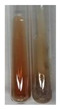	No changes were observed	–	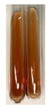	Colour change of the solution to orange-yellow	–	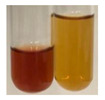
**Am Fr**	Colour change of the solution to orange-yellow	–	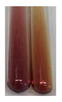	Colour change of the solution to more intense red	+	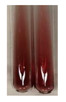	No changes were observed	–	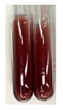	Colour change of the solution to vivid red	–	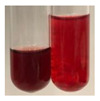
**Arv H**	Colour change of the solution to yellow	–	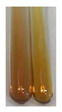	Colour change of the solution to orange	–	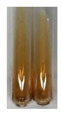	No changes were observed	–	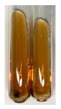	Colour change of the solution to yellow-orange; a fine precipitate formed	+	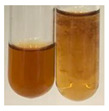
**Bv R**	Colour change of the solution to intense red	–	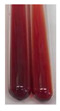	Colour change of the solution to more intense red	+	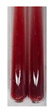	No changes were observed	–	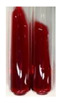	Colour change of the solution to intense raspberry	–	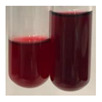
**Co F**	Colour change of the solution to intense yellow with an orange glow	–	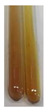	Colour change of the solution to orange with precipitation	–	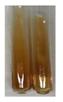	Turbidity of the solution was observed.	–	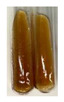	Colour change of the solution to orange; a fine precipitation formed	+	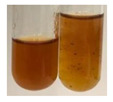
**Ea H**	Colour change of the solution to yellow-orange	–	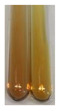	Colour change of the solution to orange-yellow	–	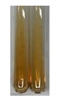	Colour change of the solution to slightly yellow	–	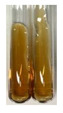	Colour change of the solution to dirty yellow	–	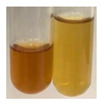
**Ep F**	Colour change of the solution to orange-yellow	–	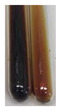	Colour change of the solution to brown-orange	–	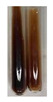	No changes were observed	–	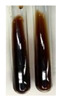	Colour change of the solution to red-orange; a red precipitate formed	–	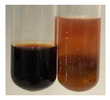
**Ep L**	Colour change of the solution to yellow-brown	–	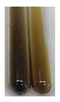	Colour change of the solution to orange-yellow	–	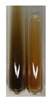	Colour change of the solution to dirty orange	–	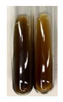	Colour change of the solution to yellow-brown	–	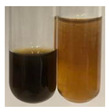
**Hp H**	Colour change of the solution to intense yellow	–	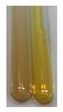	Colour change of the solution to orange	–	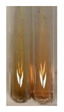	No changes were observed	–	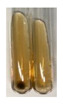	Colour change of the solution to orange; a fine precipitation formed	+	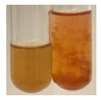
**Hr Fr**	Colour change of the solution to intense yellow	–	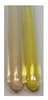	No changes were observed	–	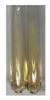	No changes were observed	–	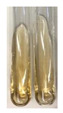	Colour change of the solution to intense yellow	–	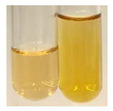
**Lc S**	Colour change of the solution to neon yellow; precipitation of a white precipitate	+	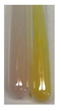	Formation of a powdery pink deposit	–	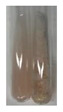	The solution became clearer	–	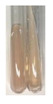	Formation of a white precipitate	–	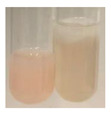
**Mc F**	Colour change of the solution to intense yellow with an orange glow	–	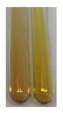	Colour change of the solution to orange-yellow	–	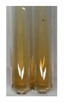	No changes were observed	–	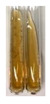	Colour change of the solution to green-yellow	–	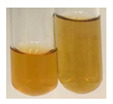
**Ob H**	Colour change of the solution to orange-yellow	–	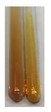	No changes were observed	–	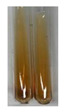	No changes were observed	–	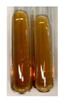	Colour change of the solution to yellow-orange; a fine precipitate formed	+	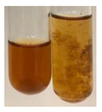
**Pm H**	Colour change of the solution to intense yellow	–	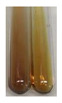	No changes were observed	–	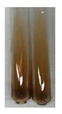	No changes were observed	–	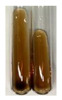	Colour change of the solution to brown	–	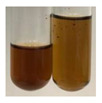
**Poa H**	Colour change of the solution to neon yellow	–	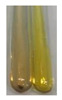	Colour change of the solution to bright orange	–	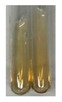	No changes were observed	–	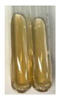	Colour change of the solution to intense yellow	–	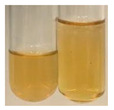
**Ps S**	Colour change of the solution to neon yellow; precipitation of a white precipitate	+	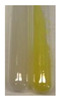	Formation of a white precipitate	–	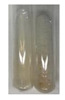	The solution became clearer	–	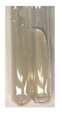	The formation of a white precipitate	+	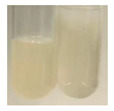
**Pta L**	The colour change of the solution to orange-yellow	–	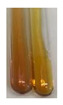	The colour change of the solution to yellow	–	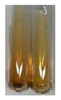	No changes were observed	–	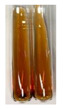	Colour change of the solution to brownish-orange; a fine precipitation formed	+	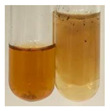
**Sg L**	Colour change of the solution to orange-yellow	–	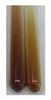	Colour change of the solution to brown-orange	+	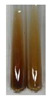	It was observed that the solution became more yellowish	–	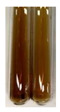	Colour change of the solution to orange-green	–	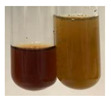
**So R**	Colour change of the solution to intense yellow; discharge of a delicate gelatinous deposit	–	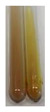	Colour change of the solution to brown-orange	+	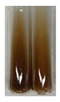	No changes were observed	–	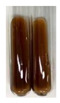	Colour change of the solution to colourless; a brown-reddish precipitate formed	+	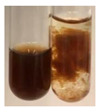
**To F**	Colour change of the solution to yellow	–	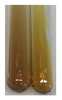	No changes were observed	–	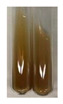	No changes were observed	–	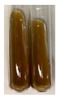	Colour change of the solution to brown-orange	–	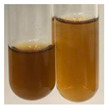
**To L**	Colour change of the solution to yellow-orange	–	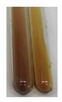	Colour change of the solution to orange	–	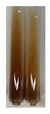	A change in degree to a brighter one was observed	–	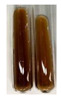	Colour change of the solution to orange; a brownish precipitate formed	+	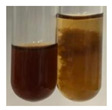
**To R**	Colour change of the solution to intense yellow	–	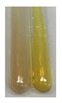	No changes were observed	–	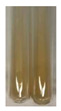	No changes were observed	–	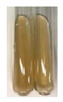	Colour change of the solution to light yellow	–	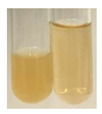
**Tp F**	Colour change of the solution to intense yellow with an orange glow	–	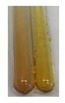	No changes were observed	–	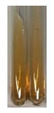	The solution became clearer	–	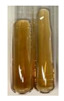	Colour change of the solution to orange-yellow	–	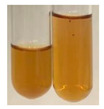
**Ur L**	The colour change of the solution to intense yellow	–	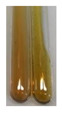	The colour change of the solution to orange	–	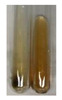	No changes were observed	–	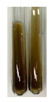	The colour change of the solution to yellow-orange	–	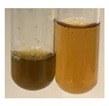
**Ur R**	Colour change of the solution to intense yellow	–	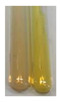	Colour change of the solution to bright orange	–	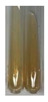	No changes were observed	–	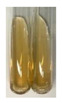	Colour change of the solution to dirty yellow	–	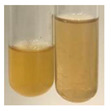
**Vo R**	Colour change of the solution to orange-yellow	–	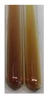	Colour change of the solution to brown	+	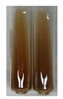	No changes were observed	–	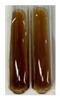	Colour change of the solution to brown-orange	–	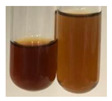

**Table 7:** The presence of alkaloids in botanical extracts–Dragendroff’s and Wagner’s tests.

Method	Dragendroff’s Test (1) and (2)			Wagner’s Test		
Extract	Observations	Result	Photograph	Observations	Result	Photograph
**Alv L**	(1) Orange solution (2) Yellow-orange solution	(1) – (2) –	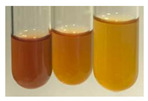	Colour change of the solution to bloody	–	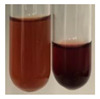
**Am Fr**	(1) Change in the shade of red colour (2) Colour change to red	(1) – (2) –	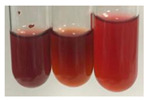	Colour change of the solution to brown-brick	–	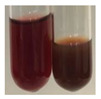
**Arv H**	(1) Brown-orange solution (2) Brown-orange solution with precipitate	(1) – (2) +	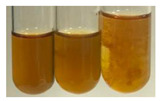	Colour change of the solution to orange-red-brown	–	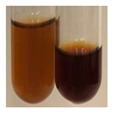
**Bv R**	(1) No changes were observed (2) No changes were observed	(1) – (2) –	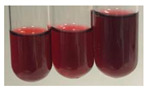	Colour change of the solution to dark-blooded	–	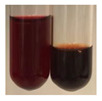
**Co F**	(1) Brown-orange solution (2) Brown-orange solution	(1) + (2) +	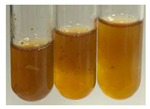	Colour change of the solution to orange-red	–	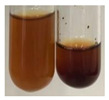
**Ea H**	(1) Orange solution (2) Orange-yellow solution	(1) – (2) –	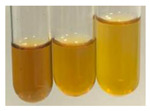	Colour change of the solution to brown-red	–	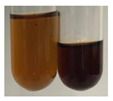
**Ep F**	(1) Brown solution (2) Orange solution + precipitation formed	(1) – (2) +	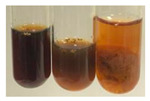	Colour change of the solution to brown-red	–	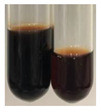
**Ep L**	(1) Cloudiness of the solution (2) Orange solution + precipitation formed	(1) – (2) +	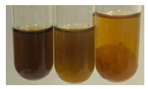	Colour change of the solution to brown	–	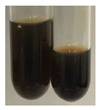
**Hp H**	(1) Orange solution (2) Orange solution	(1) – (2) –	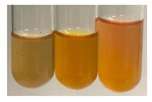	Colour change of the solution to brown	–	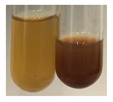
**Hr Fr**	(1) Intense yellow solution (2) Yellow solution	(1) – (2) –	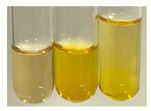	Colour change of the solution to brown	–	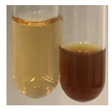
**Lc S**	(1) The formation of 2 layers: orange and cloudy white (2) The separation of 3 layers: salmon, yellow, and orange	(1) + (2) –	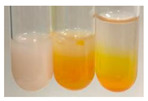	Colour change of the solution to orange	–	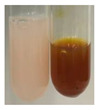
**Mc F**	(1) Yellow solution (2) Yellow solution	(1) – (2) –	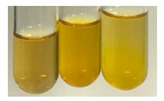	Colour change of the solution to orange-red	–	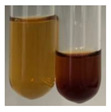
**Ob H**	(1) Brown-orange solution (2) Yellow-orange solution with fine precipitation	(1) – (2) +	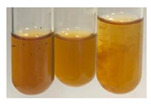	Colour change of the solution to brown-red	–	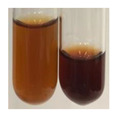
**Pm H**	(1) Brown-orange solution (2) Brown-orange solution	(1) – (2) –	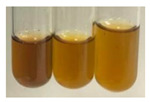	Colour change of the solution to brown	–	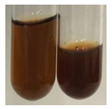
**Poa H**	(1) Yellow solution (2) The formation of 2 layers: intense yellow and lemon	(1) – (2) –	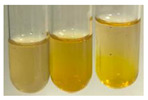	Colour change of the solution to brown-red	–	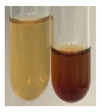
**Ps S**	(1) The formation of 2 layers: orange and light yellow (2) The formation of 3 layers: salmon, yellow, and orange (fine precipitation)	(1) + (2) +	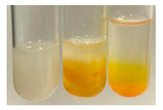	Colour change of the solution to orange	–	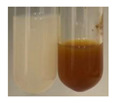
**Pta L**	(1) Orange solution (2) Yellow-orange solution	(1) – (2) –	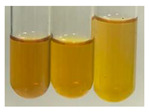	Colour change of the solution to amber; precipitation formed	+	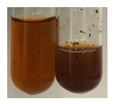
**Sg L**	(1) Brown-orange solution (2) Brown-orange solution	(1) – (2) –	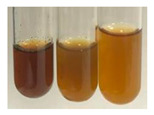	Colour change of the solution to brown-orange	–	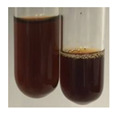
**So R**	(1) Brown-orange solution (2) Yellow-orange solution with fine precipitation	(1) – (2) +	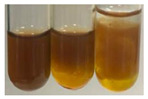	Colour change of the solution to brown	–	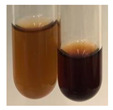
**To F**	(1) Orange-brown solution (2) Brown-orange solution	(1) – (2) –	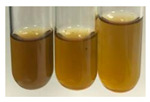	Colour change of the solution to brown-red	–	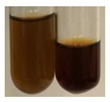
**To L**	(1) Orange-brown solution (2) Brown-orange solution with fine precipitation	(1) – (2) +	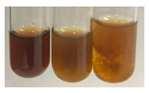	Colour change of the solution to brown	–	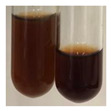
**To R**	(1) Yellow solution (2) The formation of two layers: yellow and lemon	(1) – (2) –	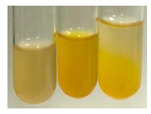	Colour change of the solution to orange-brown	–	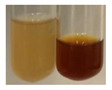
**Tp F**	(1) Orange solution (2) Orange solution	(1) – (2) –	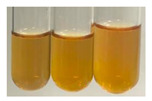	Colour change of the solution to brown-red	–	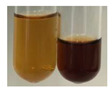
**Ur L**	(1) Brown-orange solution (2) Orange solution	(1) – (2) –	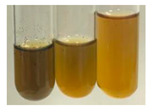	Colour change of the solution to brown-red	–	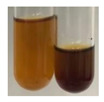
**Ur R**	(1) Orange solution (2) Orange-yellow solution	(1) – (2) –	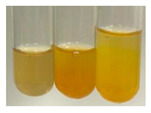	Colour change of the solution to orange-red	–	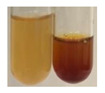
**Vo R**	(1) Brown-orange solution (2) Brown-orange solution	(1) – (2) –	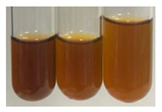	Colour change of the solution to dark brown	–	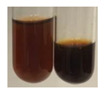

**Table 8:** The content of elements–Al, B, Ba, Ca, Cd, Cr–in raw materials (mg·kg^−1^) and botanical extracts (mg·L^−1^) (*n* = 3).

Source	Al		B		Ba		Ca		Cd		Cr	
	Raw Material	Extract	Raw Material	Extract	Raw Material	Extract	Raw Material	Extract	Raw Material	Extract	Raw Material	Extract
**Alv L**	246 ± 3	2.20 ± 0.03	15.9 ± 0.20	0.245 ± 0.005	58.6 ± 0.8	1.32 ± 0.01	37,440 ± 165	1193 ± 9	0.114 ± 0.003	<LOD	4.83 ± 0.10	0.023 ± 0.001
**Am Fr**	8.42 ± 0.17	0.030 ± 0.001	22.4 ± 0.40	0.431 ± 0.002	4.14 ± 0.16	0.024 ± 0.001	1746 ± 85	16.5 ± 0.1	0.111 ± 0.008	<LOD	2.27 ± 0.01	0.031 ± 0.002
**Arv H**	30.4 ± 0.8	0.102 ± 0.003	19.4 ± 0.20	0.214 ± 0.001	13.8 ± 0.6	0.058 ± 0.001	5992 ± 17	49.0 ± 0.3	1.84 ± 0.07	0.011 ± 0.001	0.367 ± 0.021	0.0015 ± 0.0001
**Bv R**	29.1 ± 1.2	0.060 ± 0.001	19.3 ± 0.2	0.268 ± 0.002	24.5 ± 0.7	0.199 ± 0.002	2147 ± 108	12.9 ± 0.1	0.197 ± 0.009	<LOD	0.183 ± 0.008	0.0047 ± 0.0002
**Co F**	675 ± 3	1.26 ± 0.01	52.4 ± 0.3	1.08 ± 0.01	4.69 ± 0.03	0.033 ± 0.001	5932 ± 21	84.7 ± 0.2	0.197 ± 0.005	<LOD	2.73 ± 0.05	0.081 ± 0.002
**Ea H**	37.0 ± 0.2	0.181 ± 0.002	22.7 ± 0.20	0.572 ± 0.007	22.8 ± 0.2	0.237 ± 0.004	20,815 ± 271	428 ± 1	0.123 ± 0.002	<LOD	2.42 ± 0.05	0.012 ± 0.001
**Ep F**	46.8 ± 0.4	0.965 ± 0.031	89.1 ± 0.90	1.83 ± 0.01	8.69 ± 0.0	0.140 ± 0.007	20,620 ± 31	500 ± 6	<LOD	<LOD	2.29 ± 0.03	1.84 ± 0.01
**Ep L**	62.5 ± 3.5	0.223 ± 0.011	222 ± 2	7.86 ± 0.16	26.8 ± 0.1	0.355 ± 0.002	40,254 ± 785	1234 ± 39	<LOD	<LOD	3.14 ± 0.11	0.160 ± 0.008
**Hp H**	19.5 ± 0.2	0.229 ± 0.007	20.3 ± 0.60	0.531 ± 0.005	17.9 ± 0.1	0.185 ± 0.001	3601 ± 69	75.1 ± 0.8	0.701 ± 0.017	0.0074 ± 0.0004	0.287 ± 0.02	0.0031 ± 0.0002
**Hr Fr**	18.6 ± 0.1	0.050 ± 0.002	15.2 ± 0.10	0.335 ± 0.005	0.977 ± 0.008	0.011 ± 0.001	1286 ± 11	10.5 ± 0.5	<LOD	<LOD	2.07 ± 0.04	0.014 ± 0.001
**Lc S**	6.51 ± 0.01	0.048 ± 0.002	6.56 ± 0.03	0.090 ± 0.001	1.64 ± 0.01	0.013 ± 0.001	300 ± 2	7.43 ± 0.06	<LOD	<LOD	2.64 ± 0.02	0.0076 ± 0.0004
**Mc F**	83.2 ± 1.1	0.140 ± 0.003	35.5 ± 0.4	0.749 ± 0.004	3.11 ± 0.04	0.022 ± 0.001	8061 ± 11	128 ± 1	0.524 ± 0.024	<LOD	1.79 ± 0.03	0.021 ± 0.001
**Ob H**	562 ± 8	1.66 ± 0.01	31.1 ± 0.30	0.465 ± 0.001	26.8 ± 0.3	0.276 ± 0.003	24,738 ± 210	348 ± 1	0.205 ± 0.010	<LOD	1.21 ± 0.03	0.0045 ± 0.0002
**Pm H**	334 ± 2	0.981 ± 0.006	29.8 ± 0.2	0.338 ± 0.003	52.3 ± 0.3	1.44 ± 0.01	28,384 ± 312	935 ± 2	0.210 ± 0.011	0.0037 ± 0.0001	1.42 ± 0.02	0.0055 ± 0.0003
**Poa H**	492 ± 26	1.37 ± 0.01	21.4 ± 0.20	0.181 ± 0.003	18.4 ± 0.6	0.069 ± 0.001	7326 ± 37	15.2 ± 0.2	0.376 ± 0.001	<LOD	6.08 ± 0.13	0.0066 ± 0.0002
**Ps S**	0.943 ± 0.013	0.023 ± 0.001	7.08 ± 0.06	0.104 ± 0.001	1.36 ± 0.01	0.011 ± 0.001	642 ± 17	12.8 ± 0.1	<LOD	<LOD	2.11 ± 0.05	0.0097 ± 0.0003
**Pta L**	165 ± 4	1.03 ± 0.01	26.2 ± 0.20	0.296 ± 0.004	161 ± 3.0	0.305 ± 0.002	6043 ± 21	45.4 ± 0.3	0.272 ± 0.008	<LOD	2.95 ± 0.05	0.0031 ± 0.0003
**Sg L**	50.0 ± 0.6	0.139 ± 0.007	66.7 ± 0.6	1.20 ± 0.01	13.2 ± 0.1	0.055 ± 0.001	12,182 ± 88	127 ± 1	0.144 ± 0.008	<LOD	0.314 ± 0.015	0.0037 ± 0.0001
**So R**	587 ± 6	6.08 ± 0.05	9.25 ± 0.05	0.096 ± 0.001	16.6 ± 0.1	0.205 ± 0.002	4041 ± 24	68.2 ± 0.5	0.220 ± 0.006	<LOD	1.74 ± 0.01	0.018 ± 0.001
**To F**	124 ± 6	0.309 ± 0.005	39.7 ± 0.1	0.834 ± 0.005	3.51 ± 0.06	0.032 ± 0.001	5094 ± 40	70.0 ± 1.1	0.240 ± 0.010	0.0037 ± 0.0002	0.997 ± 0.048	0.248 ± 0.004
**To L**	42.3 ± 0.4	0.148 ± 0.003	34.3 ± 0.3	0.409 ± 0.006	16.0 ± 0.2	0.127 ± 0.001	14412 ± 104	264 ± 2.6	0.685 ± 0.030	0.0057 ± 0.0001	0.269 ± 0.006	0.0076 ± 0.0003
**To R**	154 ± 1	1.51 ± 0.01	12.3 ± 0.2	0.099 ± 0.002	14.0 ± 0.2	0.089 ± 0.001	2038 ± 31	32.9 ± 0.3	0.371 ± 0.008	0.0056 ± 0.0002	1.05 ± 0.04	0.0096 ± 0.0004
**Tp F**	110 ± 2	0.311 ± 0.003	22.1 ± 0.3	0.164 ± 0.002	18.9 ± 0.3	0.151 ± 0.001	15,413 ± 170	137 ± 1	<LOD	<LOD	0.819 ± 0.016	0.011 ± 0.001
**Ur L**	98.8 ± 0.5	0.142 ± 0.003	63.3 ± 0.60	0.772 ± 0.007	41.3 ± 0.2	0.258 ± 0.001	45,964 ± 250	606 ± 3	<LOD	<LOD	2.15 ± 0.04	0.012 ± 0.001
**Ur R**	831 ± 39	1.46 ± 0.02	19.9 ± 0.90	0.115 ± 0.001	24.8 ± 0.9	0.076 ± 0.001	10,496 ± 268	43.2 ± 0.6	0.149 ± 0.006	<LOD	4.71 ± 0.22	0.0089 ± 0.0003
**Vo R**	517 ± 4	2.92 ± 0.02	16.0 ± 0.1	0.197 ± 0.001	16.5 ± 0.2	0.123 ± 0.001	2013 ± 9	29.2 ± 0.3	0.260 ± 0.003	0.0047 ± 0.0002	2.96 ± 0.027	0.013 ± 0.001

**Table 9:** The content of elements–Cu, Fe, K, Mg, Mn, Na–in raw materials (mg·kg^−1^) and botanical extracts (mg·L^−1^) (*n* = 3).

Source	Cu		Fe		K		Mg		Mn		Na	
	Raw Material	Extract	Raw Material	Extract	Raw Material	Extract	Raw Material	Extract	Raw Material	Extract	Raw Material	Extract
**Alv L**	6.52 ± 0.09	0.203 ± 0.004	360 ± 3	5.21 ± 0.04	19,791 ± 297	798 ± 9	7472 ± 43	319 ± 3	67.4 ± 1.3	2.90 ± 0.02	2634 ± 16	133 ± 1
**Am Fr**	2.97 ± 0.11	0.035 ± 0.001	19.4 ± 0.9	0.048 ± 0.001	8118 ± 100	206 ± 4	795 ± 27	13.8 ± 0.2	15.1 ± 0.2	0.246 ± 0.004	20.0 ± 0.9	0.853 ± 0.03
**Arv H**	10.6 ± 0.1	0.231 ± 0.003	52.3 ± 1.5	0.132 ± 0.001	18,407 ± 271	586 ± 3	1165 ± 20	21.0 ± 0.1	78.6 ± 3.3	1.17 ± 0.01	28.6 ± 1.9	1.18 ± 0.02
**Bv R**	5.94 ± 0.15	0.098 ± 0.001	49.4 ± 1.7	0.277 ± 0.002	31,542 ± 127	745 ± 1	2068 ± 38	44.4 ± 0.1	23.0 ± 0.8	0.269 ± 0.003	6523 ± 36	126 ± 1
**Co F**	51.1 ± 0.2	0.999 ± 0.006	720 ± 3	1.27 ± 0.01	25,872 ± 160	729 ± 7	3989 ± 12	84.1 ± 0.5	34.6 ± 0.1	0.610 ± 0.003	5918 ± 22	213 ± 1
**Ea H**	6.33 ± 0.05	0.107 ± 0.002	64.7 ± 0.6	0.125 ± 0.002	27,797 ± 473	973 ± 9	4320 ± 60	146 ± 1	37.8 ± 0.3	0.896 ± 0.014	96.8 ± 1.1	2.62 ± 0.04
**Ep F**	9.32 ± 0.06	0.319 ± 0.004	67.6 ± 0.5	0.399 ± 0.019	39,187 ± 302	1414 ± 1	2587 ± 14	71.1 ± 0.7	17.1 ± 0.2	0.455 ± 0.004	36.9 ± 0.4	1.38 ± 0.09
**Ep L**	9.42 ± 0.14	0.182 ± 0.003	92.4 ± 3.7	0.297 ± 0.003	27,762 ± 341	940 ± 30	5417 ± 80	182 ± 6	56.3 ± 0.7	0.607 ± 0.004	35.9 ± 0.5	0.760 ± 0.04
**Hp H**	9.53 ± 0.02	0.290 ± 0.007	45.0 ± 1.2	0.115 ± 0.001	7608 ± 128	283 ± 2	1364 ± 25	41.6 ± 0.5	122 ± 4	3.67 ± 0.03	42.6 ± 2.1	2.24 ± 0.12
**Hr Fr**	4.73 ± 0.03	0.068 ± 0.004	38.4 ± 0.1	0.069 ± 0.003	8467 ± 110	234 ± 2	684 ± 7	16.7 ± 0.1	11.1 ± 0.1	0.305 ± 0.008	654 ± 7	17.0 ± 0.1
**Lc S**	8.62 ± 0.06	0.234 ± 0.003	71.3 ± 0.4	1.58 ± 0.02	8702 ± 57	171 ± 1	781 ± 2	17.2 ± 0.2	13.2 ± 0.2	0.362 ± 0.005	23.0 ± 0.2	0.973 ± 0.012
**Mc F**	9.06 ± 0.09	0.149 ± 0.002	116 ± 1	0.192 ± 0.001	20,513 ± 150	645 ± 4	2804 ± 11	43.4 ± 0.5	64.4 ± 0.7	1.23 ± 0.01	619 ± 11	19.4 ± 0.1
**Ob H**	23.1 ± 0.2	0.321 ± 0.004	582 ± 5	1.34 ± 0.01	33,427 ± 401	1218 ± 16	6978 ± 84	217 ± 1	65.0 ± 0.8	1.26 ± 0.01	735 ± 11	23.4 ± 0.1
**Pm H**	8.88 ± 0.09	0.212 ± 0.002	326 ± 2	0.662 ± 0.002	23,457 ± 375	924 ± 4	6093 ± 97	244 ± 1	39.0 ± 0.3	1.38 ± 0.01	8327 ± 150	442 ± 6
**Poa H**	5.73 ± 0.13	0.064 ± 0.001	464 ± 17	0.943 ± 0.002	20,924 ± 365	576 ± 6	3013 ± 39	44.4 ± 0.5	136 ± 2	1.84 ± 0.01	97.6 ± 4.9	2.81 ± 0.03
**Ps S**	7.59 ± 0.10	0.250 ± 0.004	49.1 ± 0.3	1.44 ± 0.02	9383 ± 197	237 ± 4	1144 ± 21	28.0 ± 0.5	13.7 ± 0.3	0.410 ± 0.007	23.8 ± 0.1	1.00 ± 0.01
**Pta L**	9.13 ± 0.13	0.178 ± 0.002	166 ± 3	0.210 ± 0.002	28,725 ± 112	779 ± 2	4053 ± 30	107 ± 1	47.2 ± 0.9	0.751 ± 0.004	72.7 ± 1.3	3.33 ± 0.02
**Sg L**	6.12 ± 0.16	0.132 ± 0.001	86.9 ± 0.5	0.164 ± 0.004	22,216 ± 267	677 ± 8	2829 ± 23	53.1 ± 0.4	62.0 ± 0.4	1.88 ± 0.01	38.2 ± 0.3	1.28 ± 0.01
**So R**	27.6 ± 0.20	1.09 ± 0.01	479 ± 4	4.85 ± 0.02	28,515 ± 485	960 ± 10	906 ± 4	22.5 ± 0.2	151 ± 1	0.485 ± 0.005	1757 ± 15	57.0 ± 0.2
**To F**	12.5 ± 0.1	0.306 ± 0.004	197 ± 9	0.427 ± 0.003	26,867 ± 362	846 ± 21	2490 ± 27	56.7 ± 1.2	27.4 ± 0.9	0.537 ± 0.004	233 ± 12	6.26 ± 0.04
**To L**	9.15 ± 0.12	0.235 ± 0.004	112 ± 1	0.179 ± 0.008	39,140 ± 391	1319 ± 20	5505 ± 66	161 ± 2	123 ± 1	2.87 ± 0.03	549 ± 8	16.7 ± 0.1
**To R**	5.60 ± 0.09	0.216 ± 0.004	129 ± 1	0.964 ± 0.006	12,623 ± 215	418 ± 2	904 ± 16	21.8 ± 0.2	37.3 ± 0.3	0.974 ± 0.004	219 ± 2	6.66 ± 0.06
**Tp F**	9.07 ± 0.14	0.134 ± 0.002	128 ± 1	0.238 ± 0.001	17,333 ± 151	445 ± 3	2876 ± 40	64.0 ± 0.4	51.7 ± 0.6	1.07 ± 0.01	122 ± 2	2.94 ± 0.02
**Ur L**	7.42 ± 0.04	1.32 ± 0.01	160 ± 1	0.138 ± 0.002	17,423 ± 174	520 ± 2	8611 ± 112	206 ± 1	240 ± 1	0.747 ± 0.004	94.2 ± 0.6	1.81 ± 0.08
**Ur R**	9.20 ± 0.39	0.119 ± 0.003	657 ± 28	1.11 ± 0.01	14,074 ± 321	202 ± 1	2597 ± 34	26.8 ± 0.3	39.3 ± 1.7	0.201 ± 0.002	48.0 ± 1.3	1.19 ± 0.01
**Vo R**	5.92 ± 0.07	0.176 ± 0.002	437 ± 3	1.58 ± 0.01	15,765 ± 57	561 ± 6	2927 ± 4	83.0 ± 0.7	61.0 ± 0.3	1.58 ± 0.01	56.0 ± 0.4	3.30 ± 0.03

**Table 10:** The content of elements–Ni, P, Pb, S, Sr, Zn–in raw materials (mg·kg^−1^) and botanical extracts (mg·L^−1^) (*n* = 3).

Source	Ni		P		Pb		S		Sr		Zn	
	Raw Material	Extract	Raw Material	Extract	Raw Material	Extract	Raw Material	Extract	Raw Material	Extract	Raw Material	Extract
**Alv L**	2.51 ± 0.06	0.105 ± 0.003	1806 ± 8	69.6 ± 0.7	<LOD	NA	2725 ± 35	115 ± 1	117 ± 1	4.17 ± 0.02	14.3 ± 0.1	0.681 ± 0.007
**Am Fr**	9.68 ± 0.22	0.162 ± 0.001	1331 ± 59	31.4 ± 0.4	<LOD	NA	455 ± 34	4.53 ± 0.10	5.76 ± 0.13	0.037 ± 0.001	5.41 ± 0.21	0.093 ± 0.004
**Arv H**	1.32 ± 0.06	0.066 ± 0.003	2050 ± 36	75.0 ± 1.1	<LOD	NA	1321 ± 23	38.8 ± 0.1	8.97 ± 0.1	0.050 ± 0.001	39.5 ± 0.2	0.520 ± 0.003
**Bv R**	0.702 ± 0.009	0.073 ± 0.004	2765 ± 25	73.6 ± 0.3	<LOD	NA	1166 ± 1	28.2 ± 0.5	19.3 ± 0.5	0.126 ± 0.001	27.8 ± 0.6	0.409 ± 0.003
**Co F**	2.26 ± 0.11	0.362 ± 0.006	3793 ± 7	131 ± 1	2.83 ± 0.12	NA	2366 ± 40	67.3 ± 0.9	59.0 ± 0.5	0.788 ± 0.009	9.55 ± 0.05	0.647 ± 0.006
**Ea H**	9.45 ± 0.11	0.120 ± 0.006	2717 ± 23	87.6 ± 0.6	<LOD	NA	6809 ± 65	327 ± 1	39.2 ± 0.6	0.875 ± 0.014	31.0 ± 0.2	0.239 ± 0.001
**Ep F**	9.05 ± 0.22	4.92 ± 0.02	2772 ± 50	99.3 ± 3.8	<LOD	NA	987 ± 12	21.9 ± 0.9	23.8 ± 0.2	0.450 ± 0.002	20.3 ± 0.2	3.37 ± 0.02
**Ep L**	8.26 ± 0.19	1.27 ± 0.01	2964 ± 84	34.6 ± 0.1	2.60 ± 0.05	NA	1590 ± 40	30.3 ± 0.4	70.1 ± 0.7	1.76 ± 0.01	18.9 ± 0.1	0.469 ± 0.018
**Hp H**	1.39 ± 0.06	0.058 ± 0.002	2412 ± 18	92.6 ± 2	<LOD	NA	1430 ± 18	46.9 ± 0.7	11.7 ± 0.2	0.164 ± 0.002	39.8 ± 0.1	1.12 ± 0.01
**Hr Fr**	9.67 ± 0.26	0.082 ± 0.003	1808 ± 12	18.0 ± 0.1	<LOD	NA	1496 ± 31	20.7 ± 0.5	3.60 ± 0.04	0.036 ± 0.001	11.1 ± 0.1	0.276 ± 0.007
**Lc S**	10.8 ± 0.1	0.100 ± 0.001	3603 ± 35	86.1 ± 0.3	<LOD	NA	2013 ± 38	56.4 ± 1.4	2.02 ± 0.01	0.016 ± 0.001	37.6 ± 0.2	0.869 ± 0.004
**Mc F**	1.89 ± 0.04	0.132 ± 0.001	4960 ± 55	98.6 ± 0.5	<LOD	NA	2734 ± 44	54.5 ± 0.4	6.29 ± 0.06	0.065 ± 0.001	34.5 ± 0.4	0.337 ± 0.002
**Ob H**	1.46 ± 0.02	0.083 ± 0.004	3929 ± 22	105 ± 1	<LOD	NA	3541 ± 53	90.4 ± 1.4	177 ± 2	3.25 ± 0.03	44.0 ± 0.3	0.337 ± 0.002
**Pm H**	1.14 ± 0.05	0.055 ± 0.001	2968 ± 26	100 ± 1	1.82 ± 0.09	NA	9683 ± 55	453 ± 12	132 ± 1	4.53 ± 0.02	36.1 ± 0.2	0.686 ± 0.005
**Poa H**	7.93 ± 0.10	0.094 ± 0.001	3089 ± 19	97.3 ± 0.5	<LOD	NA	2048 ± 59	48.0 ± 0.9	21.6 ± 0.6	0.067 ± 0.001	50.1 ± 1.6	0.616 ± 0.003
**Ps S**	11.8 ± 0.20	0.085 ± 0.00	4034 ± 30	121 ± 1	<LOD	NA	1773 ± 10	56.6 ± 1.5	2.15 ± 0.01	0.018 ± 0.001	34.4 ± 0.2	1.07 ± 0.02
**Pta L**	5.33 ± 0.06	0.209 ± 0.002	2596 ± 15	49.6 ± 0.4	2.74 ± 0.02	NA	2478 ± 23	65.4 ± 1.3	37.9 ± 0.4	0.370 ± 0.004	19.6 ± 0.3	1.71 ± 0.02
**Sg L**	1.10 ± 0.05	0.096 ± 0.004	2193 ± 17	63.2 ± 0.2	<LOD	NA	1484 ± 31	16.8 ± 0.7	23.7 ± 0.1	0.138 ± 0.001	48.2 ± 0.4	0.496 ± 0.001
**So R**	2.54 ± 0.12	0.162 ± 0.008	3327 ± 33	143 ± 2	1.34 ± 0.04	NA	1155 ± 16	44.4 ± 0.4	23.2 ± 0.3	0.316 ± 0.004	28.1 ± 0.2	0.544 ± 0.004
**To F**	3.03 ± 0.06	1.08 ± 0.01	4853 ± 106	182 ± 1	<LOD	NA	2459 ± 3	54.2 ± 0.9	5.84 ± 0.11	0.061 ± 0.001	37.4 ± 0.2	0.861 ± 0.005
**To L**	1.21 ± 0.02	0.099 ± 0.001	3034 ± 27	105 ± 1	<LOD	NA	2666 ± 26	76.3 ± 1.4	19.9 ± 0.2	0.244 ± 0.003	41.5 ± 0.3	0.534 ± 0.009
**To R**	3.49 ± 0.17	0.176 ± 0.006	2646 ± 24	109 ± 1	<LOD	NA	963 ± 33	38.6 ± 0.8	19.5 ± 0.2	0.149 ± 0.001	16.6 ± 0.1	0.488 ± 0.002
**Tp F**	6.64 ± 0.12	0.212 ± 0.008	2046 ± 17	48.9 ± 0.4	1.05 ± 0.06	NA	1361 ± 35	19.3 ± 0.5	37.1 ± 0.7	0.316 ± 0.001	24.8 ± 0.3	0.374 ± 0.001
**Ur L**	7.73 ± 0.25	0.280 ± 0.011	4220 ± 24	12.6 ± 0.2	<LOD	NA	8461 ± 127	257 ± 1	81.5 ± 1.1	1.21 ± 0.01	29.8 ± 0.2	0.532 ± 0.001
**Ur R**	10.7 ± 0.2	0.074 ± 0.004	4305 ± 141	67.4 ± 0.7	1.49 ± 0.03	NA	2923 ± 142	38.2 ± 0.8	60.0 ± 3.0	0.285 ± 0.002	25.4 ± 0.8	0.162 ± 0.002
**Vo R**	3.79 ± 0.06	0.134 ± 0.003	3828 ± 50	159 ± 1	1.41 ± 0.08	NA	1852 ± 50	57.1 ± 0.5	12.3 ± 0.1	0.133 ± 0.001	27.2 ± 0.1	0.588 ± 0.004

NA–not analysed.

## Data Availability

Not applicable.
